# Transforming Endoscopic Image Classification with Spectrum-Aided Vision for Early and Accurate Cancer Identification

**DOI:** 10.3390/diagnostics15212732

**Published:** 2025-10-28

**Authors:** Yu-Jen Fang, Kun-Hua Lee, Riya Karmakar, Arvind Mukundan, Yaswanth Nagisetti, Chien-Wei Huang, Hsiang-Chen Wang

**Affiliations:** 1 Department of Internal Medicine, National Taiwan University Hospital, Yun-Lin Branch, No. 579, Sec. 2, Yunlin Rd., Douliu 64041, Taiwan; toby851072@gmail.com; 2 Department of Internal Medicine, National Taiwan University College of Medicine, No. 1 Jen Ai Rd. Sec. 1, Taipei 10051, Taiwan; 3 Department of Trauma, Changhua Christian Hospital, No. 135, Nanxiao St., Changhua County, Changhua 50006, Taiwan; 88847@cch.org.tw; 4 Department of Mechanical Engineering, National Chung Cheng University, 168, University Rd., Min Hsiung, Chiayi 62102, Taiwan; karmakarriya345@gmail.com (R.K.); arvindmukund96@gmail.com (A.M.);; 5 School of Engineering and Technology, Sanjivani University, Sanjivani Factory, Singnapur, Kopargaon 423603, Maharashtra, India; 6 Department of Electronics and Communication Engineering, Vel Tech Rangarajan Sagunthala R&D Institute of Science and Technology, No. 42, Avadi-Vel Tech Road Vel Nagar, Avadi, Chennai 600062, Tamil Nadu, India; 7 Department of Nursing, Tajen University, 20 Weixin Rd., Yanpu Township, Pingtung 90741, Taiwan; 8 Department of Medical Research, Dalin Tzu Chi Hospital, Buddhist Tzu Chi Medical Foundation, No. 2, Minsheng Road, Dalin, Chiayi 62247, Taiwan; 9 Hitspectra Intelligent Technology Co., Ltd., Kaohsiung 80661, Taiwan

**Keywords:** spectrum-aided vision enhancer, hyperspectral imaging, esophageal cancer, logistic regression, VGG16, YOLOv8, MobileNetV2, principal component analysis

## Abstract

**Background/Objective**: Esophageal cancer (EC) is a major global health issue due to its high mortality rate, as patients are often diagnosed at advanced stages. This research examines whether the Spectrum-Aided Vision Enhancer (SAVE), a hyperspectral imaging (HSI) technique, enhances endoscopic image categorization for superior diagnostic outcomes compared to traditional White Light Imaging (WLI) and Narrow Band Imaging (NBI). **Methods**: A dataset including 2400 photos categorized into eight disease types from National Taiwan University Hospital Yun-Lin Branch was utilized. Multiple machine learning and deep learning models were developed, including logistic regression, VGG16, YOLOv8, and MobileNetV2. SAVE was utilized to transform WLI photos into hyperspectral representations, and band selection was executed to enhance feature extraction and improve classification outcomes. The training and evaluation of the model incorporated precision, recall, F1-score, and accuracy metrics across WLI, NBI, and SAVE modalities. **Results**: The research findings indicated that SAVE surpassed both NBI and WLI by achieving superior precision, recall, and F1-scores. Logistic regression and VGG16 performed with a comparable reliability to SAVE and NBI, whereas MobileNetV2 and YOLOv8 demonstrated inconsistent yet enhanced results. Overall, SAVE exhibited exceptional categorization precision and recall, showcasing impeccable performance across many models. **Conclusions**: This research indicates that AI hyperspectral imaging facilitates early diagnosis of esophageal diseases, hence enhancing clinical decision-making and improving patient outcomes. The amalgamation of SAVE with machine learning and deep learning models enhances diagnostic capabilities, with SAVE and NBI surpassing WLI by offering superior tissue differentiation and diagnostic accuracy.

## 1. Introduction

Globally, EC is a considerable public health concern. The two primary histological types, squamous cell carcinoma (SCC) and adenocarcinoma (ACE), exhibit substantial differences in their incidence patterns and critical etiological factors, complicating the understanding and prevention of this illness. Their elevated mortality rate is the primary trait they share [1]. SCC is common in certain regions of Africa and East Asia, often linked to lifestyle factors such as smoking and drinking alcohol. However, ACE has become common in Western countries and is associated with Barrett’s esophagus, gastroesophageal reflux disease (GERD), and obesity. Dysphagia, unaccountable weight loss, and a chronic cough are nonspecific signs of EC that may lead to late-stage detection and poor prognosis; the overall five-year survival rate typically ranges from 15% to 25% [2]. Patients undergo contemporary treatment modalities such as chemotherapy, surgery, and radiotherapy, contingent upon the type and stage of cancer [3].

The medical imaging datasets are fundamental in enhancing EC diagnoses. The National Taiwan University Hospital Yun-Lin Branch dataset consists of 2400 images that consist of three groups, training (1800), validation (400), and test (200), that are classified into eight groups (reflecting different gastrointestinal and esophageal disorders), such as malignancy, normal tissue, staining, esophageal junction, varicose veins, duodenum, stomach, and inflammation. This dataset has been used to classify EC using machine learning and deep learning models, such as logistic regression, VGG16, the Polynomial Classifier, and YOLOv8. YOLOv8 outperformed other models in terms of object detection, whereas the other models played a role in the analysis of complicated patterns and binary categories.

Spectroscopy imaging or HSI is the examination of light’s interaction with the material under analysis. It is a hybrid approach that integrates imaging and spectroscopy. Collecting spectral data from each pixel of a two-dimensional (2-D) array detector generates a three-dimensional (3-D) database of spectral and spatial information [4]. This spatial information indicates the origin of each spectrum in the samples, enabling a more accurate analysis in relation to the environmental lighting conditions. Moreover, HSI covers a continuous spectrum of light, featuring several spectral bands and an elevated spectral resolution. Consequently, HSI can capture the spectral fluctuation under two-dimensional disparate environmental situations [5]. The spectral reflectance signature curve of the image, analyzed pixel by pixel, is displayed on the left. The RGB image has three bands in red, green, and blue wavelengths. The intensity curve of a pixel in the RGB image is positioned at the far right. It quantifies the quantity of light that a certain target or object transmits, reflects, or emits [6]. The optical imaging has also improved, as compared to the existing White Light Imaging (WLI) and Narrow Band Imaging (NBI), in hyperspectral imaging (HSI), which offers more spectrally and spatially rich information to detect disease. As summarized in the recent studies, Spectral Imaging Technology, HSI, is used in biomedical imaging to better characterize tissues using multiple wavelengths, which has enhanced diagnostic image accuracy. HSI provides continuous spectral data, and unlike WLI and NBI, which use restricted spectral ranges, this provides greater distinctiveness between normal and pathological tissues. Based on these developments, the present work proposes the Spectrum-Aided Vision Enhancer (SAVE) to transform average WLI images into hyperspectral images to improve the endoscopic image classification without the need to use a specific hardware [7].

NBI functions as a specific HSI technique by acquiring images from limited wavelength bands. The exact technique improves image attributes to provide great efficacy in medical diagnostics and other activities necessitating precise discrimination [8]. Two short-wavelength light beams at 415 nm (blue) and 540 nm (green) collaborate to improve optical images in accordance with this method [9]. Tissues permit light transmission as longer wavelengths penetrate more effectively through their structure due to scattering and absorption characteristics. Medical diagnostics significantly benefit from the application of 415 nm blue light, which clarifies superior mucosal vascular features, and the complementary 540 nm green light, which enhances the visibility of submucosal Intraepithelial Papillary Capillary Loops (IPCLs) [10]. A clear color contrast is evident in the photograph, with surface vessels depicted in brown and submucosal vessels in cyan. This approach enhances the visual differentiation between blood vessels and mucosal tissue [11]. Multiple studies indicate that NBI yields more accurate diagnoses than WLI, with respect to accuracy, sensitivity, and specificity.

This paper presents SAVE, a system that converts WLI images into HSI images using band selection to identify certain narrow bands. Multiple machine learning and deep learning architectures were developed using SAVE photos in conjunction with WLI images from EC classifications. The trained results exhibited sensitivity rates, precision levels, accuracy, and F1-score metrics for comparative analysis.

## 2. Materials and Methods

### 2.1. Dataset

The dataset from the National Taiwan University Hospital Yun-Lin Branch aims to improve the diagnosis of esophageal disorders using medical images analyzed by machine learning algorithms. The complete dataset consists of 2400 images, separated into three segments: 200 in the test set, 400 in the validation set, and 1800 in the training subset, as indicated in Table 1. Eight distinct categories are employed to classify each image, representing various gastrointestinal and esophageal diseases essential for accurate diagnosis and treatment planning. The three classifications are malignancy, indicating malignant lesions in the esophagus and crucial for malignancy detection; normal, representing healthy esophageal tissue; and staining, which reveals alterations in tissue coloration that may imply pathological processes. Other groupings include the esophageal junction, which concentrates on the area where the esophagus and stomach converge, and varicose, which emphasizes irregularities in blood vessels. The dataset includes images of the duodenum, representing the initial portion of the small intestine; the stomach, crucial for comprehensive gastrointestinal evaluations; and inflammation, indicative of inflammatory conditions impacting the esophagus.

The data utilized in this paper was collected retrospectively in the Department of Gastroenterology, Ditmanson Medical Foundation Chia-Yi Christian Hospital in 2019–2023 by use of an Olympus (Olympus Corporation, Hachioji, Tokyo, Japan) EVIS EXERA III system (CV-190 platform, GIF-HQ190 model). Images were of high quality (1920 × 1080, JPEG, sRGB) and were mostly ripped in the form of stills of the normal endoscopic videos. This prospective study was conducted in accordance with the ethical standards of the Institutional Research Board and the principles of the 1964 Declaration of Helsinki and its later amendments. The study protocol was approved by the Institutional Review Board of National Taiwan University Hospital (NTUH) (IRB No. NTUH-202410087RINA; approved on 1 January 2025). Written informed consent was waived in this study because of its retrospective, anonymized design. An initial set of more than 5000 clinically validated images was subsequently selected to yield 2400 pictures after quality control into eight esophageal disease classes, such as normal, dysplasia, and esophageal cancer. Trained annotators performed the labeling, and a senior gastroenterologist confirmed them to be clinically accurate. The augmentation methods used to address the imbalance in the classes included rotation, flipping, and contrast normalization, which ensured that the classes had equal representation when training the models. Images of realistic artifacts are deliberately incorporated in the dataset to enhance the robustness of the models and represent the variability of the clinical environment.

#### PCA

**Figure 1:**
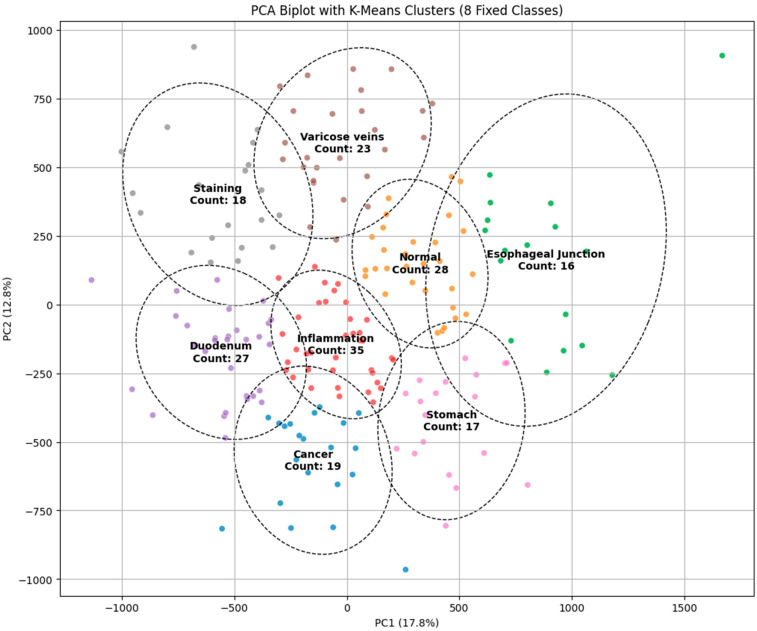
PCA biplot with K-Means clustering for classifying endoscopic images into different categories.

The principal component analysis (PCA) biplot with K-Means clustering visually represents the findings of the dimensionality reduction and clustering for the classification of endoscopic images into categories such as malignancy, inflammation, varicose veins, staining, and normal regions. The initial two principal components (PC1 and PC2) account for 17.8% and 12.8% of the variance, respectively, facilitating a distinct delineation of clusters in the diminished feature space. Distinct clusters, such as “cancer” and “normal,” display considerable separation, indicating the model’s potential for precise classification. Nevertheless, specific clusters, such as “inflammation” and “varicose veins,” exhibit significant overlap, which shows similarities in visual characteristics that could result in misdiagnosis. The cluster sizes, indicated by the number of images in each category, further highlight the data distribution, with the “varicose veins” cluster containing the most substantial sample size. The presence of overlapping regions indicates the necessity for further feature extraction methods or the incorporation of advanced classifiers to improve differentiation. The PCA visualization corroborates its efficacy by the K-Means clustering method while highlighting opportunities for enhancement to obtain more precise and dependable categorization in endoscopic image analysis, as illustrated in Figure 1.

### 2.2. Model Architecture

#### 2.2.1. Logistic Regression

Logistic regression is a fundamental statistical method utilized for medical image classification [12]. We employed logistic regression to classify EC images by analyzing grayscale image data [13]. *X* input images with dimensions 256 × 256 are initially converted into a vector of 65,536 elements. A single-layer neural network represents the logistic regression model by linearly transforming input characteristics via the weight matrix *W* and the bias term *b*, as delineated in Equation (1).

(1) Z=WX+b

The linear combination of the input features constitutes *Z* at this juncture. After the generation of the linear combination *Z*, it goes through the SoftMax activation function, yielding projected probability for all C cancer types as delineated in Equation (2).

(2) $$P\left(y_{i}=c|X\right)=\frac{e^{Z_{c}}}{\sum_{j=1}^{C}e^{Z_{j}}}$$

The input *X* is classified under class c. This probability is represented as $P\left(y_{i}=c|X\right)$. The models employs cross-entropy loss in its training process, as delineated in Equation (3).

(3) $$L=-\sum_{i=1}^{N}\sum_{c=1}^{C}y_{i,c}\log P\left(y_{i}=c|X_{i}\right)$$

The total training sample size *N*, along with *y_i_* and *c*, denotes the accurate class labels in this formula. Backpropagation calculates the gradients for Stochastic Gradient Descent (SGD) optimization, which modifies parameters *W* and *b*. The first moment estimate $m_{t}$ is calculated according to Equation (4), the subsequent moment estimate $v_{t}$ is revised in accordance with Equation (5), and the ultimate parameter adjustment is executed utilizing Equation (6).

(4) $$m_{t}=\beta_{1}m_{t-1}+(1-\beta_{1})\nabla L$$

(5) $$v_{t}=\beta_{2}v_{t-1}+\left(1-\beta_{2}\right){\left(\nabla L\right)}^{2}$$

(6) $$\theta_{t}=\theta_{t-1}-\frac{\eta }{\sqrt{\;\;\;}\;v_{t}+\epsilon }m_{t}$$

where *η* denotes the learning rate. The training spanned 300 epochs with a batch size of 64, incorporating data augmentation that used image scaling and grayscale conversion methods. The model evaluation encompassed accuracy metrics alongside confusion matrices and classification reports to ascertain its capacity to distinguish EC phases.

#### 2.2.2. VGG16

This study employed the VGG16 deep learning model to classify photos of EC. The VGG16 deep learning model operates as a convolutional neural network (CNN) pre-trained on the ImageNet dataset, making it appropriate for our research via transfer learning [14]. The fundamental form of the model utilizes small 3 × 3 kernels in convolutional layers, together with hierarchical feature extraction using max-pooling layers [15]. The extraction procedure is defined as Equation (7).

(7) $$X^{\prime }=f\left(W*X+b\right)$$

The operation includes three primary components: *X* represents the picture input, *W* denotes the filters in combination with the bias term *b*, and *f* signifies the activation function. The conclusive classification is represented by Equation (8).

(8) $$P\left(y_{i}=C|X\right)=\frac{e^{Z_{c}}}{\sum_{j=1}^{C}e^{Z_{j}}}$$

The training process utilizes the categorical cross-entropy loss function, represented as Equation (9).

(9) $$L=-\sum_{i=1}^{N}\sum_{c=1}^{C}y_{i,c}\log P\left(y_{i}=c|X_{i}\right)\;$$

The Adam optimizer was employed for optimization, utilizing the weight update rule outlined in Equations (10)–(12).

(10) $$m_{t}=\beta_{1}m_{t-1}+(1-\beta_{1})\nabla L$$

(11) $$v_{t}=\beta_{2}v_{t-1}+\left(1-\beta_{2}\right){\left(\nabla L\right)}^{2}$$

(12) $$\theta_{t}=\theta_{t-1}-\frac{\eta }{\sqrt{\;\;\;}\;v_{t}+\epsilon }m_{t}$$

The algorithm uses $m_{t}$ and $v_{t}$ as first and second moment estimations, with *η* representing the learning rate and *ϵ* serving as a safeguard against division by zero.

The training period lasted 300 epochs, incorporating data augmentation techniques such as rotation, zoom, and horizontal flipping to improve generalization. The model evaluation approach included accuracy assessment, confusion matrix analysis, and classification report examination, which demonstrated its proficiency in accurately distinguishing between different forms of esophageal cancer.

#### 2.2.3. YOLO V8

The system utilizes YOLOv8 to analyze EC while executing deep learning tasks via a controlled procedure for data management, model construction, and assessment [16]. Training commences with the confirmation of three distinct directories designated for training, validation, and testing within the dataset structure [17]. The lightweight YOLOv8n-cls.pt model is designed for classification tasks. It enhances the velocity and accuracy of its identification operations.

The training procedure spans 300 epochs, with images of 224 × 224 pixels, and does not incorporate early termination functionality due to the patience parameter being set to 0. The validation procedure initially selects data from the valid/directory, if it exists; otherwise, it defaults to the test/set data if it is valid/is devoid of data. The model is subjected to performance evaluation using the selected dataset. In classification, the cross-entropy loss serves as the primary loss function, imposing penalties for erroneous predictions, as demonstrated by Equation (13).

(13) $$L=-\sum_{i=1}^{C}y_{i}log({\hat{y}}_{i})$$

where

*C* = number of classes;

$y_{i}\;$= actual label;

${\hat{y}}_{i}$ = predicted probability for class i.

(see Supplementary Figures S13–S18)

The trained YOLOv8 model employs the test dataset for final assessment via predictions that facilitate the measurement of classification accuracy and the evaluation of the model’s stability. The processing method ensures rapid evaluation periods while maintaining precise model testing and protecting dataset features and configuration data [18].

#### 2.2.4. Mobile NetV2

The lightweight deep learning model Mobile Network Version 2 (MobileNetV2) facilitates EC classification through a structured method including dataset preparation, model training, and evaluation [19]. The data preprocessing phase performs two operations on the images: resizing each to 256 × 256 pixels and normalizing using the mean and standard deviation from ImageNet. Consequently, the model attains enhanced performance via standardized input representations [20]. MobileNetV2 attains its objective of preserving accuracy via depth-wise separable convolutions, which concurrently diminish computing complexity. The final completely linked layer utilizes C classes via the SoftMax activation function *σ*, which transforms logits into probability distributions across the C categories, as illustrated in Equation (14).

(14) $$\sigma \left(Z_{i}\right)=\frac{e^{Z_{i}}}{\sum_{j=1}^{C}e^{Z_{j}}}$$

The model optimization method relies on cross-entropy loss to assess the divergence between predicted class outcomes and actual probabilities, as delineated in Equation (15).

(15) $$L\left(y,\hat{y}\right)=-\sum_{i=1}^{C}yi\;log(({\hat{y}}_{\dot{l}})$$

The Adam optimizer updates model weights using first and second moment gradient estimates, facilitating the dynamic adjustment of the learning rate. The learning rate schedule features a step decay that reduces the learning rate by a factor of 0.1 at every 10-epoch interval. During training, AMP enables certain computations to function in 16-bit floating-point FP16 mode rather than 32-bit FP32 mode, hence enhancing performance efficiency while preserving accuracy levels [21].

The method utilizes deep learning technologies alongside optimization breakthroughs to deliver an efficient procedure for EC diagnostics, facilitating the early detection of cancer. The simultaneous implementation of the MobileNetV2 architecture, including cross-entropy loss, Adam optimization, mixed-precision training, and learning rate scheduling, enhances the model’s efficacy in medical picture classification tasks.

### 2.3. SAVE

The spectral analysis technique, in conjunction with HSI, employs band selection to identify specific wavelengths from the entire spectral range prior to further analysis. The data collection methodologies of HSI across various wavelengths yield non-informative datasets, rendering this data selection process exceedingly beneficial. SAVE was created by Hitspectra Intelligent Technology Co., Ltd., located in Kaohsiung City, Taiwan, to address band selection issues and fixed-band constraints, hence improving result accuracy. SAVE converts all RGB and WLI standard images into HSI picture format.

In spectral reconstruction inside SAVE, precise mathematical formulations facilitate the accurate transformation of images from their conventional format to hyperspectral format. The conversion calibration procedure uses the 24-color Macbeth Color Checker, incorporating natural ambient color samples. JPEG images encoded in the standard RGB (sRGB) color space necessitate a modification to standardize their R, G, and B values within the range of 0 to 1. Through linearized RGB data in conjunction with the Gamma function, the technique produces results in the CIE 1931 XYZ color space, which delineate numerical correlations between wavelengths of the visible spectrum and perceived color responses.

The conversion transforms the normalized RGB values into XYZ values (XYZcamera), hence yielding CIE XYZ tristimulus values. The camera system operates to replicate the visual representations of colors found in the Macbeth Color Checker, which serves as the reference standard. The conversion employs the specific mathematical pattern illustrated in Equations (16)–(18).

Conversion of RGB to CIE 1931 XYZ color space

(16) $$L^{*}=116f\left(\frac{Y}{Y_{n}}\right)-16$$

(17) $$a^{*}=500[f(\frac{X}{X_{n)}}-f\left(\frac{Y}{Y_{n}}\right)]$$

(18) $$b^{*}=200[f\left(\frac{Y}{Y_{n}}\right)-f\left(\frac{Z}{Z_{n}}\right)]$$

where $f\left(n\right)$ is defined in Equation (19):

(19) $$f\left(n\right)=\left\{\begin{array}{cc} n^{1/3}, & n>0.008856\\ 7.787n+0.137931, & otherwise \end{array}\right.$$

The acquired spectral data is transformed utilizing both the ophthalmoscope light source spectrum S(λ) and the XYZ color matching tool. R(λ) (380 nm–780 nm, 1 nm) within the XYZ color gamut pertains to the light source spectrum of the ophthalmoscope hyperspectral system. The procedure applies S(λ) in conjunction with the XYZ color matching algorithm.

Modifications must be implemented in the camera’s nonlinear response utilizing a third-order formula that employs $V_{Non-Linear}$ variables as response modifiers. The V matrix is derived from the standardized outputs of $V_{Color}$ and $V_{Non-Linear}$ and contains the $V_{Dark}$ variable. The standardization process is limited to the third order to prevent excessive rectification.

The color difference analysis utilizing CIEDE 2000 necessitates the transformation of both XYZ Correct and XYZ Spectrum via a translation process. An array (R(λ))401*24 was utilized to arrange the spectra in accordance with a matrix structure, wherein the matrix comprises intensity values at 1 nm wavelength intervals along its rows.

The Macbeth Color Checker comprises 24 color samples, which form the columns in this configuration.

**Figure 2:**
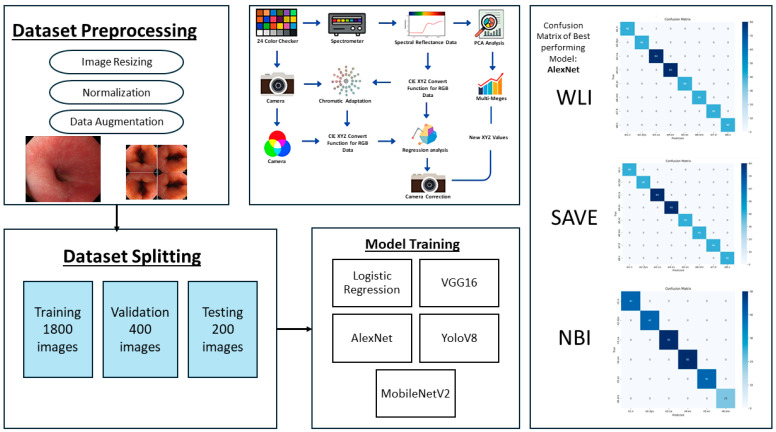
Workflow of the proposed framework, showing dataset preprocessing, splitting, model training, color correction pipeline, and confusion matrices of the best performing model for WLI, SAVE, and NBI.

The established method ensures flawless spectrum conversion, hence improving the accuracy rate and operational efficiency of endoscopic HSI. As illustrated in Figure 2, the study followed a structured pipeline starting with preprocessing and dataset partitioning, followed by model training with different architectures. A color correction step was incorporated to improve image consistency, and model performance was assessed.

**Figure 3:**
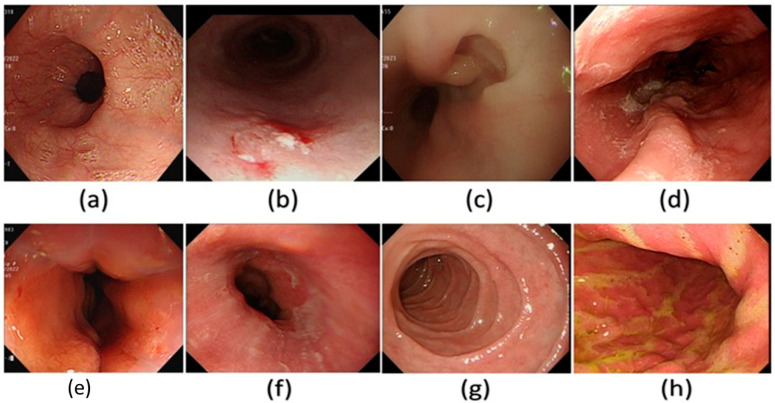
A few examples of the WLI images used in the dataset in this study. (**a**) Normal, (**b**) dysplasia, (**c**) cancer, (**d**) varicose veins, (**e**) esophageal junction, (**f**) inflammation, (**g**) duodenum, and (**h**) stomach.

**Figure 4:**
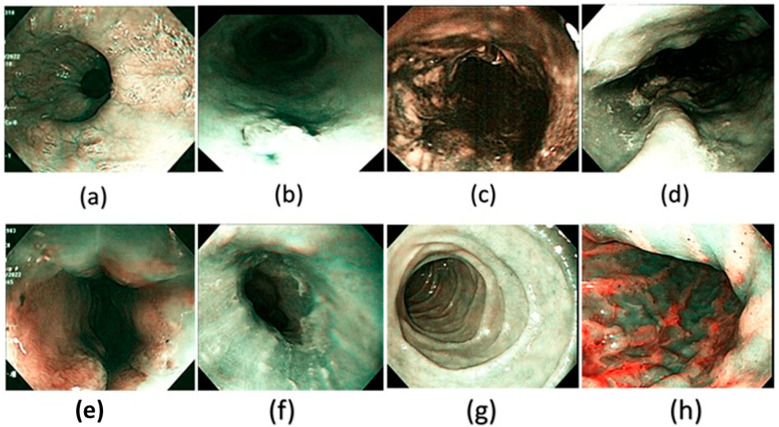
A few examples of the corresponding SAVE images of the WLI generated and used in the dataset in this study. (**a**) Normal, (**b**) dysplasia, (**c**) cancer, (**d**) varicose veins, (**e**) esophageal junction, (**f**) inflammation, (**g**) duodenum, and (**h**) stomach.

**Figure 5:**
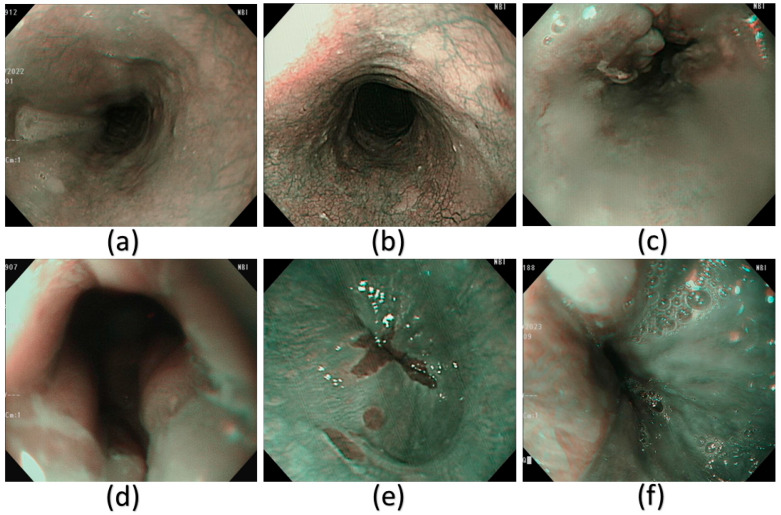
A few examples of the NBI images that have been used in this study. (**a**) Normal, (**b**) dysplasia, (**c**) cancer, (**d**) esophageal junction, (**e**) inflammation, and (**f**) varicose veins.

Spectral reconstruction of SAVE technique is performed by creating a fine correlation between RGB or images of white light (WLI) as shown in Figure 3 and reference spectral information with the Macbeth Color Checker (X-Rite Classic) to calibrate it as shown in Figure 4 (see supplementary Table S2 for the RMSEs of the XYZ values before and after calibration and Table S3 for the color difference before and after camera calibration). It is a color checker with a set of 24 standardized color patches that represent natural colors, which is used in measurements when converting endoscopic RGB images to the CIE 1931 XYZ color space. The values in the RGB color space are scaled and linearized with Gamma correction function to ensure that the responses to colors are true to life. The corrected values are then remapped to values of the XYZ tristimulus values (XYZ camera), such that SAVE can reproduce the spectral behavior of the reference Macbeth chart. SAVE identifies the most informative spectral features and removes the redundant data using multiple regression (MRA) and principal component analysis (PCA). The PCA-based band selection is an eigenvector-based band selection method, which reveals the eigenvectors with the highest contribution to the spectral variance, or, in other words, more than 99% of information, thus dimensionality reduction is achieved without compromising diagnostic fidelity. This guarantees that only necessary wavelength bands are kept, improving computational efficiency and retaining important spectral properties required to make medical imaging produce true color and tissue in medical imaging (see supplementary Figure S29 for the RMSEs between analog and measured spectra of each color block; Figure S30 for the chosen hyperspectral bands for the SAVE algorithm and Figure S31 for the SSIM and PSNR test for the SAVE algorithm). The WLI images shown in Figure 3 and the corresponding SAVE images as shown in Figure 4 are compared with the original NBI images shown in Figure 5.

## 3. Results

### 3.1. Logistic Regression

The research data in Table 2 shows that WLI, SAVE, and NBI demonstrate dissimilation efficacy in esophageal condition diagnosis. WLI faces data classification challenges in two primary domains: normal tissue identification and staining tissue recognition. A considerable percentage of standard cases that require classification are erroneously detected by the algorithm, indicating a recall rate of 0.45. The accuracy of the “staining” detection is 1.00, while its precision is 0.62, indicating a robust identification of staining but resulting in erroneous classifications of other disease categories (as depicted in Supplementary Figure S1 for training and validation loss/accuracy and in Supplementary Figure S2 for the confusion matrix on WLI). WLI exhibits robust efficacy in diagnosing both “cancer” and “varicose veins,” characterized by high precision and recall values, while also attaining exceptional outcomes for “inflammation.” The identification accuracy rate for the “esophageal junction” is 0.82, indicating the presence of some erroneous classifications.

SAVE exhibits total success in category categorization, as its precision, recall, and F1-scores attain perfect values of 1.00. The accuracy rate of SAVE indicates flawless functioning, since it does not make erroneous conclusions regarding any condition category. The superior reliability of SAVE renders it more precise than WLI, as SAVE consistently identifies each condition without misclassifications, as illustrated in Supplementary Figure S3 for the confusion matrix and Supplementary Figure S4 for the training and validation performance on SAVE images. SAVE represents an advanced imaging technique because of its exceptional precision in classifying esophageal conditions.

The classification performance of NBI exhibits flawless accuracy, since its precision score, recall score, and F1-score each attain a value of 1.00 for every class. Both NBI and SAVE showed remarkable efficacy in condition discrimination, as evidenced by the absence of misclassifications (seen in Supplementary Figure S5 for the confusion matrix and corroborated in Supplementary Figure S6 for training and validation performance on NBI images). The advanced imaging techniques SAVE and NBI surpass WLI in efficacy by producing enhanced diagnostic information via an increased imaging contrast.

This research indicates that SAVE, in conjunction with NBI, surpasses WLI as a more effective tool for categorizing esophageal tissue patterns. SAVE and NBI have attained impeccable diagnostic accuracy, demonstrating an exceptional performance that positions both procedures as superior to WLI.

### 3.2. VGG16

The performance outcomes presented in Table 3 indicate that WLI, SAVE, and NBI yield distinct performance metrics for the VGG16 model, encompassing accuracy, precision, recall, and F1-scores. The WLI system provides a 97% accuracy, whereas SAVE and NBI attain a slight advantage with a 99% accuracy. The classification reliability metrics from SAVE and NBI surpass those generated by WLI, as seen in Supplementary Figure S7 for the training/validation performance and Supplementary Figure S8 for the confusion matrix of WLI images.

WLI has commendable precision and recall scores across the majority of categories, with little fluctuations in performance. The “normal” category of WLI exhibits a precision of 0.90 and a recall of 0.95, although it does not attain the exact scores observed in both the SAVE and NBI systems. The recall for “staining” is 0.97, accompanied with an accuracy value of 0.89, indicating a minor degree of misclassification. WLI demonstrates flawless performance in the “duodenum” and “stomach” as well as the “cancer” category, achieving a total precision and recall of 1.00. The F1-scores throughout the categories remain continuously elevated, while slight variations indicate occasional misclassifications.

SAVE achieves flawless classification across six spatial pathology categories, with the precision, recall, and F1-score all at 1.00 for “staining,” “cancer,” “esophageal junction,” “inflammation,” “duodenum,” and “stomach.” The model exhibits a flawless capability to identify these circumstances without generating any errors. The classification of “varicose veins” exhibits a recall score of 0.96, falling short of the optimal perfect rating. SAVE exhibits remarkable dependability as an imaging technique for classification, with a total accuracy of 99% (as illustrated in Supplementary Figure S9 for the confusion matrix and in Supplementary Figure S10 for the training and validation performance on SAVE images).

NBI achieves exceptional outcomes with its nearly flawless precision metrics, recall metrics, and F1-score assessments, which generally maintain entire accuracy levels. The “normal” group along with “staining” and “varicose veins” have nearly perfect F1-scores of 0.97, 0.98, and 0.99, respectively. The “cancer” classification attains flawless precision (1.00) alongside a recall of 0.98, indicating few erroneous negative classifications. NBI demonstrates its efficacy in diagnosing esophageal problems, achieving a 99% accuracy in performance, as illustrated in Supplementary Figure S11 for the confusion matrix and in Supplementary Figure S12 for training and validation accuracy trends on NBI images.

### 3.3. YOLOV8

The YOLOv8 model exhibits varying performance metrics across WLI, SAVE, and NBI modalities, revealing distinct strengths and weaknesses in the analysis of different classes, as illustrated in Table 4. The model demonstrates a mid-range performance relative to VGG16, as it does not achieve optimal classification results, as shown in Supplementary Figure S19 for the WLI confusion matrix and Supplementary Figure S20 for detection outputs on SAVE images.

The WLI findings demonstrate that the model achieves an 81% accuracy, with F1-scores for various classes ranging from 0.69 to 0.95. The duodenum and stomach classes exhibit superior performance with an F1-score of 0.95, alongside elevated precision and recall scores; however, inflammation shows a diminished F1-score of 0.69, while the esophageal junction attains an F1-score of 0.78. The model exhibits difficulty in distinguishing between normal and staining situations, since both achieve an F1-score of 0.71, as further corroborated by Supplementary Figure S21, which illustrates classification results on NBI images.

The model’s application on SAVE achieves an accuracy rate of 85%. The F1-scores surpass WLI measurements throughout many classes, as the duodenum attains a flawless score of 1.00, while the stomach and cancer categories, together with varicose veins, earn robust F1-scores of 0.86 and 0.91, respectively. The model encounters comparable difficulties in identifying inflammation (0.71 F1-score) and the esophageal junction (0.80 F1-score) as it does with WLI. The issues caused by SAVE have a minimal impact on predictions; nonetheless, its superior capacity to distinguish specific classes improves its reliability, as illustrated in Supplementary Figure S22, which illustrates YOLOv8’s multiclass confusion matrix.

The NBI task achieved an accuracy rate of 82%, exhibiting an inferior performance compared to SAVE yet surpassing WLI in results. The F1-score indicates that staining cases have an accuracy of 0.95, whereas cancer and normal cases attain F1-scores of 0.98 and 0.83, respectively. The F1-score reflects diminished efficacy in classifying inflammation (0.53) and the esophageal junction (0.74). The identification of varicose veins via the model results in an F1-score of 0.71, whereas other conditions exhibit superior accuracy, as illustrated in Supplementary Figure S23 for precision–recall trade-offs and corroborated in Supplementary Figure S24 with an additional confusion matrix reflecting YOLOv8 performance trends. The model results suggest that NBI provides minimal supplementary advantages relative to SAVE for the diagnostic task.

### 3.4. MobileNetV2

The MobileNetV2 model demonstrates superior performance with WLI as the imaging technique, with an accuracy of 86.5%, whereas SAVE yields an 80% accuracy, and NBI results in the lowest performance at 75%, as depicted in Table 5. MobileNetV2 encounters challenges with classification types other than those it can accurately identify, specifically excluding inflammation or the esophageal junction, as demonstrated in Supplementary Figure S25, which displays the WLI confusion matrix, and in Supplementary Figure S26, which presents the associated training and validation performance.

The WLI accuracy rate of MobileNetV2 reaches 86.5%, with an F1-score of 0.86 for normal cases, 0.92 for varicose veins, 0.97 for the duodenum, and 0.98 for stomach inspections. The classification effectiveness of the model markedly declines for inflammation and staining, as evidenced by F1-scores of 0.68 and 0.76, respectively. The WLI method exhibits improved performance compared to other strategies for this model system.

The model’s accuracy with SAVE diminishes to 80%, and F1-scores decrease across several categories. Wireless endoscopy exhibits an acceptable classification ability, achieving F1-scores over 0.81 for normal cases, the duodenum, stomach regions, varicose veins sections, and cancer tissues. SAVE demonstrates suboptimal category classification for staining and inflammation, yielding an F1-score of 0.64, and for the esophageal junction, with an F1-score of 0.77, due to diminished accuracy in these domains (shown in Supplementary Figure S27, which presents the confusion matrix for NBI pictures).

The accuracy measurement of NBI is 75%, the lowest result among MobileNetV2’s imaging approaches. Staining and varicose veins achieved robust classification results with F1-scores of 0.87 and 0.80, respectively; however, inflammation attained just a 0.27 F1-score, while the esophageal junction recorded a 0.67 F1-score. The low F1-score for classifying inflammation indicates that it is a hard condition for NBI, as the technique exhibits poor accuracy, as evidenced by Supplementary Figure S28 for the training and validation metrics of NBI images.

**Figure 6:**
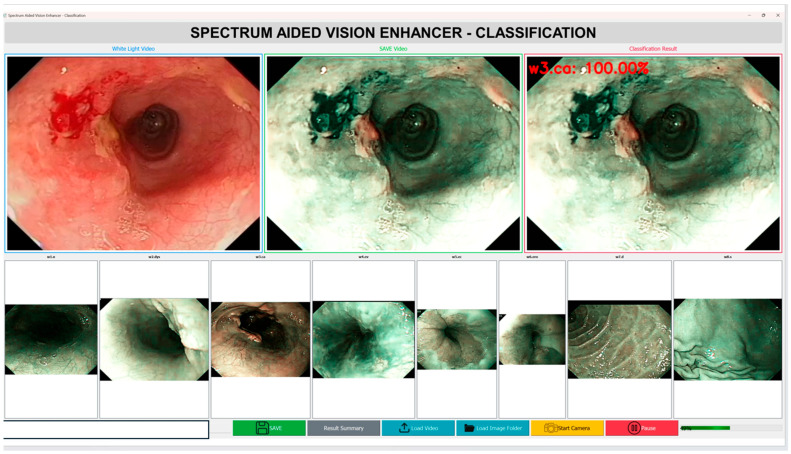
Computational efficiency and inference speed of the Spectrum-Aided Vision Enhancer (SAVE).

In addition to the accuracy of the diagnostics, the computational efficiency and the inference speed of the Spectrum-Aided Vision Enhancer (SAVE) framework are critical factors, which determine its feasibility in the context of real-time implementation in the clinical setting as shown in Figure 6. To test the feasibility of the end-to-end performance of SAVE in a practical operating environment, a prototype application has been created as part of this pilot study. With a typical video sequence of 1440 frames with 24 frames per second (fps) and a White Light Imaging (WLI) video sequence, the entire hyperspectral conversion took about 135 s on a workstation with a current-day GPU. This translates to an average processing of 10.6 fps, which means that SAVE runs at a close to real-time speed with standard computational hardware. The latency seen is largely due to the per frame hyperspectral reconstruction and feature extraction steps that are aimed at preserving the spectral integrity and diagnostic accuracy. Being a pilot study, these results confirm the technical feasibility of SAVE and point to the areas for optimization. In future studies, our work will be aimed at improving the computational throughput by using light-weighted neural architectures and parallelization on a GPU level, and after that, the frame rate may be reduced to 12 fps to achieve further optimizations of the real-time operating efficiency. Overall, this pilot study indicates that SAVE is a viable tool for future large-scale real-time endoscopic diagnostic systems that can be used in clinical settings to provide high-quality and quick interpretations of hyperspectral images. A two-factor analysis of variance (ANOVA) without replication was used to assess the performance differences among imaging modalities and assessment metrics among all models (see Supplementary Table S1 for the Anova results). This study revealed a significant overall effect (F(15, 30) = 11.13, *p* = 1.98 × 10^−8^), indicating substantial heterogeneity in categorization methodologies and performance metrics. Nonetheless, the imaging modality did not have a statistically significant influence (F(2, 30) = 2.39, *p* = 0.1088 > 0.05, F_ncrit_ *n* = 3.32). Nevertheless, a significant numerical trend emerged, with SAVE achieving the highest overall performance (91.19 ± 9.76), followed by WLI (89.05 ± 7.12) and NBI (87.66 ± 12.67). The results demonstrate that SAVE consistently outperformed standard modalities in terms of its mean precision, recall, and F1-score; however, the differences were not statistically significant given the current sample size. The experiment’s results, incorporating additional data and reduced inter-model variance, require further examination to confirm the statistical superiority of SAVE.

## 4. Discussion

This research paper discusses the application of machine learning and deep learning technologies for the interpretation of medical images to classify esophageal disorders. This study illustrates the critical necessity of the timely detection of EC, since this health concern is a significant global public health challenge [22]. Advanced imaging technology combined with machine learning models enhances endometrial cancer analysis and treatment planning due to the disease’s dismal survival rates [23].

This study included 2400 medical images sourced from Kaohsiung Medical University, categorized into eight unique classes encompassing diverse esophageal and gastrointestinal diseases. An appropriate allocation of images into training, validation, and testing segments facilitated a comprehensive model evaluation. The disease classification encompassed cancer, normal tissue, staining, varicose veins, esophageal junction abnormalities, inflammation, and diseases of the duodenum and stomach [24]. This study assessed various machine learning and deep learning frameworks to optimize classification accuracy [23].

This study included a combination of logistic regression alongside VGG16, YOLOv8, and MobileNetV2. The primary statistical model of logistic regression executed binary tasks, while both CNNs and VGG16 accomplished image recognition functions [25]. This research chose YOLOv8 for its rapid object detection capabilities and MobileNetV2 for its efficient lightweight architecture, making it suitable for medical imaging applications [26].

This study presented SAVE, a novel technique designed to convert WLI images into HSI via its established transformation method. The research team assessed the performance of WLI, NBI, and SAVE imaging modalities. The research findings indicated that SAVE, in conjunction with NBI, had a superior performance compared to WLI in the assessment of illness categories. SAVE attained a flawless classification performance, establishing it as a promising instrument for medical image analysis. The precision, recall, and F1-score attained a value of 1.00 for all classes.

The evaluation of the model performance revealed that VGG16 attained complete success in image categorization, with a 100% accuracy rate across all techniques. The performance of YOLOv8 differed by its imaging approach, with WLI achieving an 81% accuracy, SAVE attaining 85%, and NBI realizing 82% success. The optimal outcome from MobileNetV2 was achieved with WLI images, resulting in an accuracy of 86.5%. The accuracy rates of SAVE and NBI were 80% and 75%, respectively. Research demonstrates that deep learning models effectively detect diseases; nevertheless, their detection performance is contingent upon the chosen imaging approach [27].

The assessment of machine learning models necessitates the accurate evaluation of four fundamental metrics: the precision, recall, F1-score, and accuracy [28]. This work demonstrates that hyperspectral imaging combined with NBI is crucial for enhancing diagnostic accuracy in the detection of esophageal disorders [29]. This research facilitates medical imaging advancement by integrating sophisticated imaging systems with deep learning models, thereby generating new opportunities for the early diagnosis of esophageal conditions [30].

### 4.1. Practical Challenges

The SAVE algorithm is very much dependent on an accurate calibration of the endoscopic camera and the spectrometer with 24 colors using the Macbeth Color Checker. But this calibration accuracy in the real world is difficult to maintain. Spectral sensitivities and nonlinear characteristics of different endoscope models, light sources, and camera sensors differ. As an example, variations in the illumination spectra, gamma correction, and dark current may corrupt the information on the color recorded and create imprecisions in the reconstructed hyperspectral data. Furthermore, optical properties of biological tissues, including scattering, absorption, and reflection, are different across patients and body parts. These inconsistencies complicate the attainment of the spectral-to-color mapping of different imaging sessions. As a result, a small calibration error will be magnified by several steps of transformation, which decreases the accuracy of simulated fine-band images. The SAVE algorithm consists of numerous computational steps that are computationally expensive, which consist of a conversion to color space, PCA, regression modeling, spectral reconstruction, and NBI simulation. All of these stages require great processing power and memory bandwidth. Whereas these kinds of computations can be handled offline using high-performance workstations, the merger of SAVE with real-time medical imaging systems is a significant issue. There are strict limitations on the size, power consumption, and processing capacity of capsule endoscopes as well as portable video endoscopes. To achieve clinical frame rates of 2530 fps, special hardware acceleration is required to achieve real-time execution. In the absence of this, latency and frame drops might interfere with live visualization in the course of diagnostic or surgical processes. Furthermore, heterogeneity exists between the hardware of various manufacturers, and so a universal SAVE-compatible platform is not easily possible. One of the biggest problems with the implementation of SAVE is the validation of the simulated NBI images, in particular with the VCE. There is a native NBI mode in traditional Olympus endoscopes that can be compared directly with simulated results, whereas VCEs do not have such an option. The lack of a reference ground truth makes it hard to determine the degree of the fidelity of SAVE to the actual NBI appearance and diagnostic characteristics. Although such measures as SSIM, PSNR, and entropy demonstrate a quantitative similarity, they have no guarantees of being clinically interpretable or lesion-visible. Moreover, the training and evaluation data is quite limited, and it might not reflect the extensive stochastic range of the mucosal texture, vascular patterns, and pathologies in practice. Thus, prior to the clinical implementation of SAVE, it is necessary to carry out an extensive validation with larger, multi-center datasets and use expert samples to ensure diagnostic reliability.

### 4.2. Advantages and Limitations of Imaging Modalities

The imaging modalities have their own strengths and weaknesses that affect the success of the analysis of medical images. The most widely used modality in endoscopic diagnostics is still WLI because it is widely available, naturally colored, and cheap to compute. It allows for the rapid acquisition of images and can work with normal clinical processes. However, WLI has a poor contrast and spectral information, and, therefore, it has a low sensitivity for early lesion detection and faint tissue differentiation [31]. Conversely NBI helps improve the visualization of the mucosal structures and vascular patterns with the help of narrow-band light with the central wavelengths of 415 nm and 540 nm, which are the hemoglobin absorption peaks. This enhances the detection of vascular and neoplastic anomalies, and NBI is useful in the early diagnosis of esophageal and gastrointestinal cancers. However, its significant shortcomings are that it utilizes fixed spectral bands and that it also relies on special optical filters, which are costly equipment and restrict the spectral flexibility necessary to fully characterize tissues [32]. The proposed SAVE provides a computational alternative, which is able to rebuild the hyperspectral images using normal RGB or WLI pictures, to generate more information on spectral images, without having to use any specific optical equipment. This enables a better differentiation of the tissues and a high level of diagnostic accuracy, especially when identifying abnormalities, which depend on the characteristics of hemoglobin absorption. Nevertheless, SAVE also adds more complexity and requires the high-end calibration of colors to guarantee spectral fidelity between systems of various endoscopes. These issues are critical in achieving rational and ubiquitous clinical integration [31].

Each machine learning and deep learning model employed in the present study has its own strengths and weaknesses that affect its applicability to the medical imaging. Logistic regression is highly interpretable and low in computational costs and hence best suited to small datasets or grayscale image analysis. Nonetheless, it is a linear model by nature and, therefore, cannot represent nonlinear relationships that are complex in high-dimensional image data, explaining its lower accuracy in the context of WLI images as raw data [32]. The VGG16 model is better at the task of image classification, showing accuracies of up to 99–100 percent after being trained on SAVE-processed images or NBI images. This has been attributed to its high hierarchical feature extraction and strong ability to extract finer details of images. Its large computational and parameter size is the main trade-off, as this could limit its use in real-time or resource-constrained clinical settings [33].

YOLOv8 offers a good trade-off between speed and accuracy, as it is fast and accurate when it comes to real-time detection and classification in the field of live endoscopy. It features powerful spatial localization due to its anchor-free nature and efficient pyramid of features [34]. It can however be less sensitive to small spectral differences, because the model is specifically designed to be sensitive to spatial features rather than spectral or textural features. Finally, MobileNetV2 is designed to be deployed on small and embedded platforms and has much lower computational demands. It has a moderate classification accuracy. Although its accuracy is less than that of more deep convolutional networks, it is still acceptable for resource-constrained applications. In order to achieve clinically reliable performance rates, MobileNetV2 might need spectral augmentation or spectral SAVE-based improvement, which offers more discriminative features. In general, these models when combined with SAVE demonstrate potential for effective, precise, and real-time medical image analysis with a variety of hardware and clinical conditions [34].

## 5. Conclusions

Machine learning and deep learning methodologies have demonstrated efficacy in assessing medical images of esophageal disorders, underscoring the importance of timely and precise diagnoses to improve treatment outcomes. The assessment of various classification models, including logistic regression, VGG16, YOLOv8, and MobileNetV2, applied to WLI, NBI, and SAVE images was conducted using data from Kaohsiung Medical University. The study findings indicated that NBI and SAVE exhibited enhanced disease detection capabilities compared to WLI, with SAVE achieving a flawless classification performance for each disease category tested. VGG16 achieved the highest results with 100% accuracy, whereas other algorithms exhibited varying performances depending on the chosen imaging method. This research indicates that AI-driven diagnostic tools, when combined with HSI, exhibit significant potential for the improved diagnosis of esophageal diseases. Deep learning systems utilizing advanced imaging techniques enhance medical diagnostic capabilities, resulting in faster and more accurate diagnoses of esophageal diseases. Future research must improve deep learning models by expanding data gathering and incorporating supplementary imaging techniques to enhance diagnostic accuracy. The integration of AI diagnostic technologies into medical practice holds significant potential for transforming the recognition and treatment of esophageal diseases, resulting in enhanced healthcare outcomes.

## Figures and Tables

**Table 1:** Class-wise distribution in gastrointestinal endoscopic image dataset.

Class	Train	Validation	Test	Total
Normal	180	40	20	240
Staining	180	40	20	240
Cancer	360	80	40	480
Varicose Veins	360	80	40	480
Esophageal Junction	180	40	20	240
Inflammation	180	40	20	240
Duodenum	180	40	20	240
Stomach	180	40	20	240
Total	1800	400	200	2400

**Table 2:** Performance metrics of logistic regression for classifying esophageal conditions across WLI, SAVE, and NBI modalities.

Type	Classes	Precision	Recall	F1-Score	Accuracy
WLI	Normal	1.00	0.45	0.62	92%
	Staining	0.62	1.00	0.77	
	Cancer	1.00	0.94	0.97	
	Varicose veins	1.00	0.93	0.96	
	Esophageal junction	0.82	1.00	0.90	
	Inflammation	1.00	1.00	1.00	
	Duodenum	1.00	1.00	1.00	
	Stomach	1.00	1.00	1.00	
SAVE	Normal	1.00	1.00	1.00	100%
	Staining	1.00	1.00	1.00	
	Cancer	1.00	1.00	1.00	
	Varicose veins	1.00	1.00	1.00	
	Esophageal junction	1.00	1.00	1.00	
	Inflammation	1.00	1.00	1.00	
	Duodenum	1.00	1.00	1.00	
	Stomach	1.00	1.00	1.00	
NBI	Normal	1.00	1.00	1.00	100%
	Staining	1.00	1.00	1.00	
	Cancer	1.00	1.00	1.00	
	Varicose veins	1.00	1.00	1.00	
	Esophageal junction	1.00	1.00	1.00	
	Inflammation	1.00	1.00	1.00	

**Table 3:** VGG16 deep learning model classification results for esophageal diseases using WLI, SAVE, and NBI techniques.

Type	Classes	Precision	Recall	F1-Score	Accuracy
WLI	Normal	0.90	0.95	0.93	97%
	Staining	0.89	0.97	0.93	
	Cancer	1.00	1.00	1.00	
	Varicose veins	0.99	0.95	0.97	
	Esophageal junction	1.00	0.97	0.99	
	Inflammation	1.00	0.95	0.97	
	Duodenum	1.00	1.00	1.00	
	Stomach	1.00	1.00	1.00	
SAVE	Normal	0.93	1.00	0.96	99%
	Staining	1.00	1.00	1.00	
	Cancer	1.00	1.00	1.00	
	Varicose veins	1.00	0.96	0.98	
	Esophageal junction	1.00	1.00	1.00	
	Inflammation	1.00	1.00	1.00	
	Duodenum	1.00	1.00	1.00	
	Stomach	1.00	1.00	1.00	
NBI	Normal	0.97	0.97	0.97	99%
	Staining	0.95	1.00	0.98	
	Cancer	1.00	0.98	0.99	
	Varicose veins	1.00	0.98	0.99	
	Esophageal junction	1.00	1.00	1.00	
	Inflammation	1.00	1.00	1.00	

**Table 4:** YOLOv8 object detection model performance on esophageal disease classification using WLI, SAVE, and NBI images.

Type	Classes	Precision	Recall	F1-Score	Accuracy
WLI	Normal	0.68	0.75	0.71	81%
	Staining	0.75	0.68	0.71	
	Cancer	0.86	0.86	0.86	
	Varicose veins	0.92	0.81	0.86	
	Esophageal junction	0.80	0.76	0.78	
	Inflammation	0.75	0.64	0.69	
	Duodenum	0.95	0.95	0.95	
	Stomach	0.95	0.95	0.95	
SAVE	Normal	0.80	0.84	0.82	85%
	Staining	0.75	0.71	0.73	
	Cancer	0.83	0.90	0.86	
	Varicose veins	0.97	0.86	0.91	
	Esophageal junction	0.80	0.80	0.80	
	Inflammation	0.85	0.61	0.71	
	Duodenum	1.00	1.00	1.00	
	Stomach	1.00	1.00	1.00	
NBI	Normal	0.90	0.76	0.83	82%
	Staining	0.95	0.95	0.95	
	Cancer	0.96	1.00	0.98	
	Varicose veins	0.64	0.80	0.71	
	Esophageal junction	0.80	0.70	0.74	
	Inflammation	0.50	0.56	0.53	

**Table 5:** MobileNetV2 classification accuracy and metrics for esophageal diseases using WLI, SAVE, and NBI modalities.

Type	Classes	Precision	Recall	F1-Score	Accuracy
WLI	Normal	0.79	0.95	0.86	86.5%
	Staining	0.73	0.80	0.76	
	Cancer	0.81	0.85	0.83	
	Varicose veins	1.0	0.85	0.92	
	Esophageal junction	0.90	0.90	0.90	
	Inflammation	0.72	0.65	0.68	
	Duodenum	1.00	0.95	0.97	
	Stomach	0.95	1.00	0.98	
SAVE	Normal	0.84	0.80	0.82	80%
	Staining	0.53	0.80	0.64	
	Cancer	0.84	0.78	0.81	
	Varicose veins	0.80	0.80	0.80	
	Esophageal junction	0.79	0.75	0.77	
	Inflammation	0.73	0.55	0.63	
	Duodenum	1.00	0.95	0.97	
	Stomach	0.95	1.00	0.98	
NBI	Normal	0.80	0.80	0.80	75%
	Staining	0.89	0.85	0.87	
	Cancer	0.83	0.76	0.79	
	Varicose veins	0.80	0.80	0.80	
	Esophageal junction	0.57	0.80	0.67	
	Inflammation	0.40	0.20	0.27	

## Data Availability

The original contributions presented in this study are included in the article/Supplementary Materials. Further inquiries can be directed to the corresponding authors.

## Supplementary Materials

The following supporting information can be downloaded at: https://www.mdpi.com/article/10.3390/diagnostics15212732/s1, refs. [14,21,35,36,37,38,39,40,41,42,43,44,45] are cited in the Supplementary Materials. Table S1: Anova Analysis for the three imaging modalities of WLI, NBI and SAVE; Table S2: RMSEs of the XYZ values before and after calibration; Table S3: The color difference before and after camera calibration; Figure S1: Training and Validation loss and accuracy for Logistic regression; Figure S2: Confusion matrix illustrating classification performance of logistic regression on WLI dataset; Figure S3: Confusion Matrix depicting logistic regression classification accuracy on SAVE images; Figure S4: Training and Validation loss and accuracy of SAVE images for Logistic regression; Figure S5: Confusion matrix for Logistic Regression performance on NBI dataset; Figure S6: Training and Validation loss and accuracy of NBI images for Logistic regression; Figure S7: Training and Validation loss and accuracy of WLI images for VGG16 model; Figure S8: Confusion matrix for VGG16 classifier on WLI images indicating True Positive Rate vs. False Positive Rate; Figure S9: Confusion matrix for VGG16 model evaluated on SAVE imaging data for multiclass esophageal classification; Figure S10: Training and Validation loss and accuracy of SAVE images for VGG16; Figure S11: Confusion matrix reflecting VGG16 classification output on NBI images; Figure S12: Training and Validation loss and accuracy of NBI images for VGG16; Figure S13: Confusion matrix for YOLOv8 object detection model on WLI images; Figure S14: Detection output visualization of YOLOv8 model on SAVE images indicating Region-Based classification; Figure S15: YOLOv8 output on NBI Images showing detection accuracy and localization of esophageal abnormalities; Figure S16: Confusion matrix summarizing YOLOv8 classification accuracy; Figure S17: Precision-Recall curve of YOLOv8 model demonstrating detection trade-offs across thresholds; Figure S18: Confusion matrix performance of YOLOv8 model; Figure S19: Confusion matrix displaying MobileNetV2 performance on WLI images for esophageal condition classification; Figure S20: Training and Validation loss and accuracy of WLI images for MobileNetv2 model; Figure S21: Confusion Matrix reflecting MobileNetV2 predictions on NBI images; Figure S22: Training and Validation loss and accuracy of NBI images for MobileNetv2 model; Figure S23: Training and Validation loss and accuracy of WLI images for AlexNet model; Figure S24: Confusion matrix of AlexNet classifier Applied to WLI Images with Class-Wise evaluation; Figure S25: Training and Validation loss and accuracy of SAVE images for AlexNet model; Figure S26: Confusion matrix of AlexNet classifier applied to SAVE images with Class-Wise evaluation; Figure S27: Training and Validation loss and accuracy of NBI images for AlexNET model; Figure S28: Confusion matrix for AlexNet applied to NBI images highlighting model prediction strengths and weaknesses; Figure S29: RMSEs between analog and measured spectra of each color block; Figure S30: The chosen hyperspectral bands for the SAVE algorithm; Figure S31: The SSIM and PSNR test for the SAVE algorithm.
