# Supplementary material for: Transforming Endoscopic Image Classification with Spectrum-Aided Vision for Early and Accurate Cancer Identification
Source: Diagnostics (Basel). 2025 Oct 28;15(21):2732. doi: 10.3390/diagnostics15212732 (PMC12607974; doi:10.3390/diagnostics15212732)
Supplement: Supplementary file 1 [file diagnostics-15-02732-s001.zip › diagnostics-3887302-supplementary.pdf]

diagnostics

MDPI

Article

Transforming Endoscopic Image Classification with Spectrum-Aided Vision for Early and Accurate Cancer Identification: Supplementary

Yu-Jen Fang [1,2], Kun-Hua Lee [3,4], Riya Karmakar [4,5], Arvind Mukundan [4,5], Yaswanth Nagisetti [6], Chien-Wei Huang [7,8,*], and Hsiang-Chen Wang [4,9,*]

[1] Department of Internal Medicine, National Taiwan University Hospital, Yun-Lin Branch, No. 579, Sec. 2, Yunlin Rd., Dou-Liu 64041, Taiwan; toby851072@gmail.com

[2] Department of Internal Medicine, National Taiwan University College, Department of Internal Medicine, National Taiwan University College of Medicine, No. 1 Jen Ai Rd. Sec. 1, Taipei 10051, Taiwan

[3] Department of Trauma, Changhua Christian Hospital, Changhua; No.135, Nanxiao St., Changhua City, Changhua County, 50006, Taiwan, 88847@cch.org.tw (K.-H.L.)

[4] Department of Mechanical Engineering, National Chung Cheng University, 168, University Rd., Min Hsiung, Chia Yi 62102, Taiwan; karmakarriya345@gmail.com (R.K.); arvindmukund96@gmail.com (A.M.)

[5] School of Engineering and Technology, Sanjivani University, Sanjivani Factory, Singnapur, Kopargaon, Maharashtra 423603, India

[6] Department of Electronics and Communication Engineering, Vel Tech Rangarajan Dr.Sagunthala R&D Institute of Science and Technology, No.42, Avadi-Vel Tech Road Vel Nagar, Avadi, Chennai, Tamil Nadu 600062, India; vtu19659@veltech.edu.in

[7] Department of Nursing, Tajen University, 20, Weixin Rd., Yanpu Township, Pingtung County 90741, Taiwan.

[8] Department of Medical Research, Dalin Tzu Chi Hospital, Buddhist Tzu Chi Medical Foundation, No. 2, Minsheng Road, Dalin, Chiayi, 62247 Taiwan

[9] Director of Technology Development, Hitspectra Intelligent Technology Co., Ltd., Kaohsiung 80661, Taiwan

[*] Correspondence: forevershiningfy@yahoo.com.tw (C.-W.H.); hcwang@ccu.edu.tw (H.-C.W.)

Academic Editor: Firstname Lastname

Received: date
Revised: date
Accepted: date
Published: date

Citation: To be added by editorial staff during production.

Copyright: © 2025 by the authors. Submitted for possible open access publication under the terms and conditions of the Creative Commons Attribution (CC BY) license (https://creativecommons.org/licenses/by/4.0/).

Abstract: Background/Objective: Esophageal cancer (EC) is a major global health issue due to its high mortality rate, as patients are often diagnosed at advanced stages. This research examines whether the Spectrum-Aided Vision Enhancer (SAVE), a hyperspectral imaging (HSI) technique, enhances endoscopic image categorization for superior diagnostic outcomes compared to traditional White Light Imaging (WLI) and Narrow Band Imaging (NBI). **Methods:** A dataset including 2400 photos categorized into eight disease types from Kaohsiung Medical University was utilized. Multiple machine learning and deep learning models were developed, including Logistic Regression, VGG16, YOLOv8, and MobileNetV2. SAVE was utilized to transform WLI photos into hyperspectral representations, and band selection was executed to enhance feature extraction and improve classification outcomes. The training and evaluation of the model incorporated precision, recall, F1-score, and accuracy metrics across WLI, NBI, and SAVE modalities. **Results:** The research findings indicated that SAVE surpassed both NBI and WLI by achieving superior precision, recall, and F1-scores. Logistic Regression and VGG16 performed solid reliability with SAVE and NBI, whereas MobileNetV2 and YOLOv8 demonstrated inconsistent yet enhanced results. Overall, SAVE exhibited exceptional categorization precision and recall, showcasing impeccable performance across many models. **Conclusion:** The research indicates that based on AI hyperspectral imaging facilitates early diagnosis of esophageal diseases, hence enhancing clinical decision-making and improving patient outcomes. The amalgamation of SAVE with machine learning and deep learning models enhances diagnostic capabilities, with SAVE and NBI surpassing WLI by offering superior tissue differentiation and diagnostic accuracy.

Models Evaluation

The precision and recall and F1-Score metrics are derived from the confusion matrix through the following calculation methods using TP, FP, FN and TN values.

1. Precision (Positive Predictive Value)

The precision rate determines the number of correctly identified instances among all the predicted positive cases [1] as shown in Equation 1.

$$Precision = \frac{TP}{TP+FP} \quad (1)$$

- A correct prediction of positive instances results in a True Positive outcome.
- Successful predictions of true positives and inaccurate predictions of positive cases are denoted by TP (True Positives) and FP (False Positives) [2].

2. Recall (Sensitivity or True Positive Rate)

Recall determines the percentage of actual positive cases which the model correctly identifies [3]. The Equation 2 shows formula for calculating recall.

$$Recall = \frac{TP}{TP+FN} \quad (2)$$

These wrong predictions dismissed instances that should have been categorized positively (False Negatives)

3. F1-Score

The F1-score computes as the harmonic average between Precision and Recall scores which offers balanced measurement when class distributions are unbalance [4] as shown in Equation 3.

$$F1 - Score = 2 \times \frac{Precision \times Recall}{Precision + Recall} \quad (3)$$

- The F1-score helps achieve balanced results when precision and recall measurement needs adjustment especially for situations where one value is much lower than the other.

4. Accuracy

The accuracy metric counts all predictions to determine how many were correct with respect to total predictions [5]. The formula of accuracy is mentioned in Equation 4.

$$Accuracy = \frac{TP+TN}{TP+TN+FP+FN} \quad (4)$$

- This measure identifies positive instances correctly when the prediction returns negative results [6].
- Accuracy functions well with balanced datasets yet provides incorrect signals when dealing with unbalanced datasets.

Logistic Regression

Overview:

Logistic Regression is a statistical learning machine that is common in binary and multiclass classification. It was used in this research with grayscale image of esophageal cancer to develop a baseline model against deep learning architectures[7].

Preprocessing:

256x256 pixel images were reduced to grayscale, and flattened into 65,536-elements one-dimensional arrays. Every feature vector was brought to a normalized range (0, 1) to normalize the input distribution and enhance the optimization. Random resizing as well as intensity normalization were also used to augment the dataset.

Model Structure:

Logistic Regression is a single-layer neural network that changes the input features X into a linear equation:

$$\boldsymbol{Z} = \boldsymbol{WX} + \boldsymbol{b} \quad (1)$$

where W denotes the weight matrix and b the bias term. The output Z is run through a SoftMax activation function to give class probabilities:

$$\boldsymbol{P}(\boldsymbol{y_i} = \boldsymbol{c}|\boldsymbol{X}) = \frac{e^{Z_c}}{\sum_{j=1}^{C} e^{Z_j}} \quad (2)$$

Loss Function and Optimization:

The categorical cross-entropy loss, which is minimized by the model, is:

$$\boldsymbol{L} = -\sum_{i=1}^{N} \sum_{c=1}^{C} \boldsymbol{y_{i,c}} \log \boldsymbol{P}(\boldsymbol{y_i} = \boldsymbol{c}|\boldsymbol{X_i}) \quad (3)$$

Optimization was accomplished using Stochastic Gradient Descent (SGD) supplemented with the Adam update method, as shown:

$$m_t = \beta_1 m_{t-1} + (1 - \beta_1)\nabla L \quad (4)$$

$$v_t = \beta_2 v_{t-1} + (1 - \beta_2)(\nabla L)^2 \quad (5)$$

$$\theta_t = \theta_{t-1} - \frac{\eta}{\sqrt{v_t + \epsilon}} m_t \quad (6)$$

where η is the learning rate. The training lasted for 300 epochs with a batch size of 64 under data augmentation conditions that included image resizing procedures together with grayscale conversion techniques. The model evaluation included accuracy measures in addition to confusion matrices and classification reports to establish its ability in differentiating esophageal cancer stages.

Convergence and Evaluation:

Training and validation loss was used to monitor the training process. The convergence was decided at the point the magnitude of change in loss between successive epochs was smaller than 10^{-6}. Accuracy, confusion matrices and classification reports were used to assess model performance[8].

VGG16

Overview:

VGG16 structure is a deep convolutional neural network (CNN) that was created by the Visual Geometry Group at Oxford. It is simple in nature, with small convolution filters (3×3) stacked in deep sequential layers. VGG16 was utilized in this work to extract the features and classify the esophageal cancer images via feature extraction through transfer learning[9].

Preprocessing:

The input images were scaled to 224x224x3 and normalized with the values of ImageNet mean and standard deviation (mean = [0.485, 0.456, 0.406]; std = [0.229, 0.224, 0.225]). Random rotations, zooming and horizontal flipping were added to the data to augment its robustness.

Network Architecture:

It has 13 convolutional layers, 5 max-pooling layers, and 3 fully connected layers, which have a total of about 138 million parameters. The convolutional operation is as follows[9]:

$$X' = f(W * X + b) \quad (7)$$

Conv is used to represent the convolution operation and f is the ReLU activation function. The last layer uses SoftMax function to generate probability distribution among all classes:

$$P(y_i = C|X) = \frac{e^{Z_c}}{\sum_{j=1}^{C} e^{Z_j}} \quad (8)$$

Loss and Optimization:

The model uses categorical cross-entropy loss and Adam optimizer to optimize the model using gradient-based optimization with a learning rate of 0.001. Adam updated rule can be classified as:

$$m_t = \beta_1 m_{t-1} + (1 - \beta_1)\nabla L \quad (10)$$

$$v_t = \beta_2 v_{t-1} + (1 - \beta_2)(\nabla L)^2 \quad (11)$$

$$\theta_t = \theta_{t-1} - \frac{\eta}{\sqrt{v_{t+\epsilon}}} m_t \quad (12)$$

The number of epochs was 300, and to speed up the convergence, they were trained with the help of batch normalization that enhances the stability.

Convergence and Evaluation:

Convergence was dictated by the plateau of the validation loss and saturation of the accuracy. Accuracy, confusion matrix and classification measures were used to evaluate the final model performance.

YOLOv8

Overview:

YOLOv8 is a state of the art, anchor free convolutional neural network that is capable of detecting objects and classifying images in real time. Here, the YOLOv8 was modified to classify images of esophageal cancer, and it utilizes its high performance and speed. Preprocessing: The data was divided into training, validation and test folders. All the images were brought to 224×224 pixels and scaled to the range [0,1]. Augmentation used was mosaic augmentation, mix-up, random flipping, and hue-saturation variation augmentation to augment generalization[10].

Model Architecture:

YOLOv8 is designed as a system with three primary components that consist of the backbone, the neck, and the head. The backbone deals with the multiscale feature extraction and makes use of Cross Stage Partial (CSP) blocks to effectively extract meaningful features of input pictures. The neck, designed on the Path Aggregation Network (PANet), integrates and boosts the features of diverse resolutions to generate the model to be sensitive to detect the objects of varied sizes. Lastly, the head guesses the class likelihood and bounding box coordinates, so that precise object localization and classification is made possible[12]. In this analysis, the YOLOv8n-cls.pt model was utilized, which is more suitable as a classification task.

Loss Function and Optimization:

The Cross-Entropy Loss is minimized in the model:

$$L = -\sum_{i=1}^{C} y_i \log(\hat{y}_i) \quad (13)$$

The AdamW optimizer that combines the advantages of adaptive learning rate adjustment and the weight decay regularization was used to train. The learning rate was at 0.001 and the epochs of training were 300.

Convergence and Evaluation:

The convergence criterion was founded on the stabilization of validation losses and mean Average Precision plateau. In the model, the classification performance was assessed in terms of accuracy, precision, recall, and F1-score, which proved the reliability and efficiency of the model in cancer image classification to be high[11].

MobileNetV2

Overview:

MobileNetV2 is a model of a deep learning model that targets embedded and mobile devices. It was applied in this research to offer a computationally effective but an accurate approach to image classification of esophageal cancer[7].

Preprocessing:

Each image was scaled to 256 x 256 x 3 pixels and scaled with ImageNet mean and standard deviation to have all features scaled in similar manner. Randomized rotations, flipping and zooming of the data augmented the data to minimize overfitting.

Model Architecture:

MobileNetV2 uses depth wise separable convolutions and inverted residual blocks in order to minimize computation and representational power. Each block consists of Feature expansion by a 1×1 pointwise convolution,3×3 depth convolution based in spatial filtering, and. A linear dimensionality reduction (1x1)[13].

In case the input and output dimensions are equal, residual connections are added. The last layer is a fully connected layer which produces class logits which are transformed to probabilities to use SoftMax activation:

$$\boldsymbol{\sigma}(\boldsymbol{Z_i}) = \frac{\boldsymbol{e^{Z_i}}}{\sum_{j=1}^{C} \boldsymbol{e^{Z_j}}} \qquad (\mathbf{14})$$

Loss Function and Minimization:

The cross-entropy loss is employed in the model:

$$L(y, \hat{y}) = -\sum_{i=1}^{C} yi \log(\hat{y}_i) \qquad (15)$$

Adam optimizer with a step decay learning rate schedule was used to optimize it with a learning rate that is decreased by a factor of 0.1 after every 10 epochs. Automatic Mixed Precision (AMP) training was also turned on which enables the operations to be run in FP16 mode to achieve better performance and memory efficiency[13].

Convergence and Evaluation:

The convergence was calculated using the loss stabilization and validation accuracy. The training lasted 300 epochs, and the measures of the evaluation were accuracy, precision, recall, and F1-score.

MobileNetV2 proved highly classification efficient and greatly lowered the burden of computation and was thus appropriate when performing medical image analysis on low-resource systems.

Experimental Environment and Training Parameters

All the experiments were carried out in a controlled computational environment such that consistency and reproducibility were maintained across models. The program setup contained Python 3.10 as the programming language, and the main deep learning frameworks were PyTorch 2.2.0 and TensorFlow 2.15. This was implemented using OpenCV 4.8, scikit-image 0.22, and NumPy 1.26 to perform image preprocessing and enhancement operations and Matplotlib 3.8 to do result analysis and visualization. Pandas 2.2 was used to handle and load data and the CUDA Toolkit 12.3 and cuDNN libraries 9.0 were used to accelerate the process with the use of a single GPU.

Training of each model was done using a common set of hyperparameters so that results can be compared. The images were scaled to 256 x 256 and done in batches of 64 samples at a time. The optimization algorithm used to train was Adam, the initial learning rate was 0.001, momentum values were 0.9 and 0.999, and the step decay scheduler lowered the learning rate by a factor of 0.1 after every 10 epochs. Cross-entropy loss function has been used as the main optimization criterion in all classification tasks. To enhance the generalization of the model, various data augmentation algorithms were used, such as random rotations (±15), zoom (±10%), flipping left and right, and adjustment of brightness (±5%). The training dataset was split into 20 percent validation to track the performance of the models and avoid overfitting in all the experiments.

Each deep learning architecture, namely, VGG16, YOLOv8, and MobileNetV2 were pre-trained on ImageNet weights and then fine-tuned using the SAVE-transformed dataset as well as the original dataset to adjust the learned representations to the esophagus cancer classification problem. Conversely, the Logistic Regression was applied with the help of grayscale image features as baseline. Every experiment was done thrice to obtain reproducibility and reliability and reported metrics of the performance reflect the mean values of these three independent runs. Precision, recall, F1-score, and accuracy were the key indicators of performance that were used to evaluate the final outcome as a way of giving a very viable evaluation of the classification effectiveness of all models.

S.no	Before calibration			After calibration			RMSE	SD
	X	Y	Z	X	Y	Z		
1	10.96	9.92	4.63	11.14	9.87	4.26	0.24	0.30
2	38.74	35.80	18.65	38.57	35.94	18.66	0.13	0.08
3	16.62	19.07	24.13	16.48	18.79	24.11	0.18	0.17
4	10.33	12.86	4.62	10.16	13.03	4.85	0.19	0.19
5	24.05	23.87	31.55	24.16	24.07	31.60	0.13	0.08
6	30.12	42.15	32.40	30.10	42.17	32.42	0.02	0.002
7	38.10	30.24	4.28	38.04	30.37	4.22	0.09	0.04
8	11.70	11.47	25.90	11.64	11.37	25.91	0.07	0.02

9	29.01	19.91	9.62	29.20	19.78	9.60	0.13	0.08
10	8.26	6.49	9.63	8.06	6.49	9.86	0.18	0.17
11	34.15	44.06	8.44	34.15	44.02	8.53	0.06	0.01
12	47.99	44.55	6.05	48.05	44.34	6.17	0.15	0.11
13	6.82	5.79	21.07	6.90	5.91	21.00	0.09	0.04
14	14.55	23.55	7.22	14.58	23.51	7.12	0.07	0.02
15	21.08	12.25	3.57	21.01	12.28	3.65	0.06	0.01
16	58.40	60.69	7.54	58.38	60.79	7.42	0.09	0.04
17	28.98	19.54	20.67	28.94	19.52	20.66	0.02	0.002
18	12.81	19.01	28.54	12.84	19.10	28.56	0.05	0.01
19	82.12	88.54	67.20	82.31	88.73	67.51	0.24	0.30
20	54.74	58.92	45.52	54.28	58.40	44.75	0.60	1.89
21	33.08	35.73	27.24	33.26	35.82	27.54	0.21	0.23
22	18.18	19.62	14.94	18.86	20.31	15.62	0.68	2.43
23	9.13	10.01	8.13	8.56	9.26	7.21	0.76	3.04
24	2.87	3.19	2.39	3.10	3.35	2.68	0.23	0.27
Average							0.19	0.39

Table S1. RMSEs of the XYZ values before and after calibration.

S.no	Before Camera Calibration	Spectrometer	Chromatic Aberration	After Camera Calibration	Spectrometer	Chromatic Aberration
1			7.08			1.24
2			7.63			0.78
3			16.43			0.86
4			12.45			1.68

5			14.92			0.45
6			10.80			0.05
7			7.47			0.52
8			18.46			0.22
9			13.19			0.62
10			8.09			1.30
11			8.03			0.09
12			6.43			0.58
13			10.32			0.30
14			12.19			0.23
15			13.31			0.17
16			7.00			0.18
17			17.80			0.03
18			22.22			0.19
19			0.00			0.08
20			5.30			0.30
21			9.77			0.42
22			12.71			0.81
23			13.34			2.01
24			3.37			1.96
Average Color Difference			10.76	Average Color Difference		0.63

Table S1. The color difference before and after camera calibration

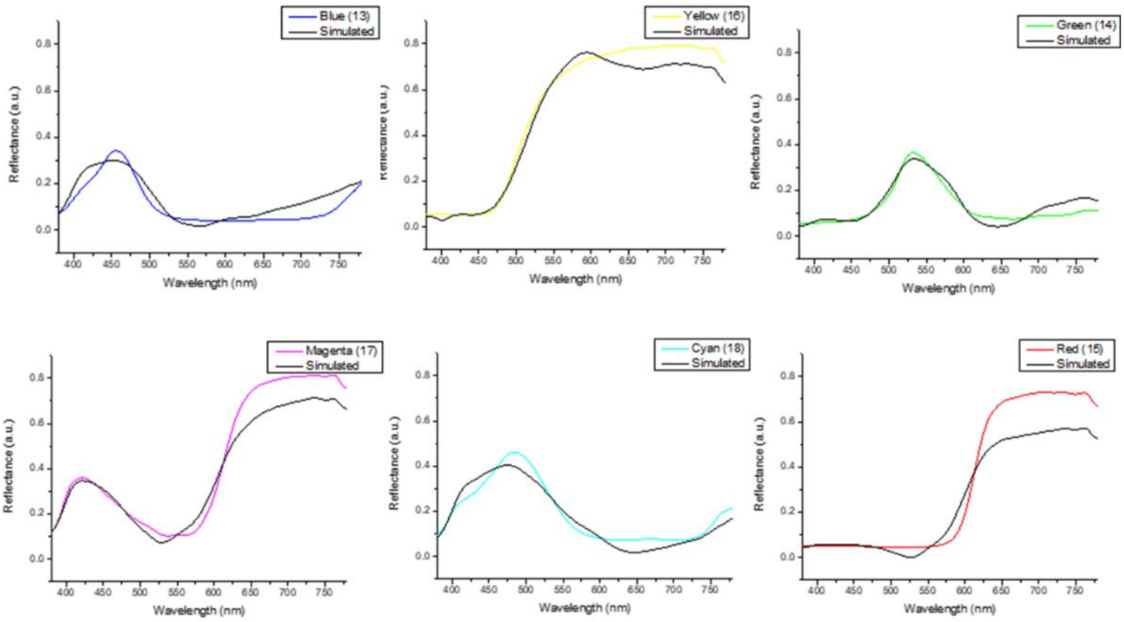

Figure Table S3. RMSEs between analog and measured spectra of each color block

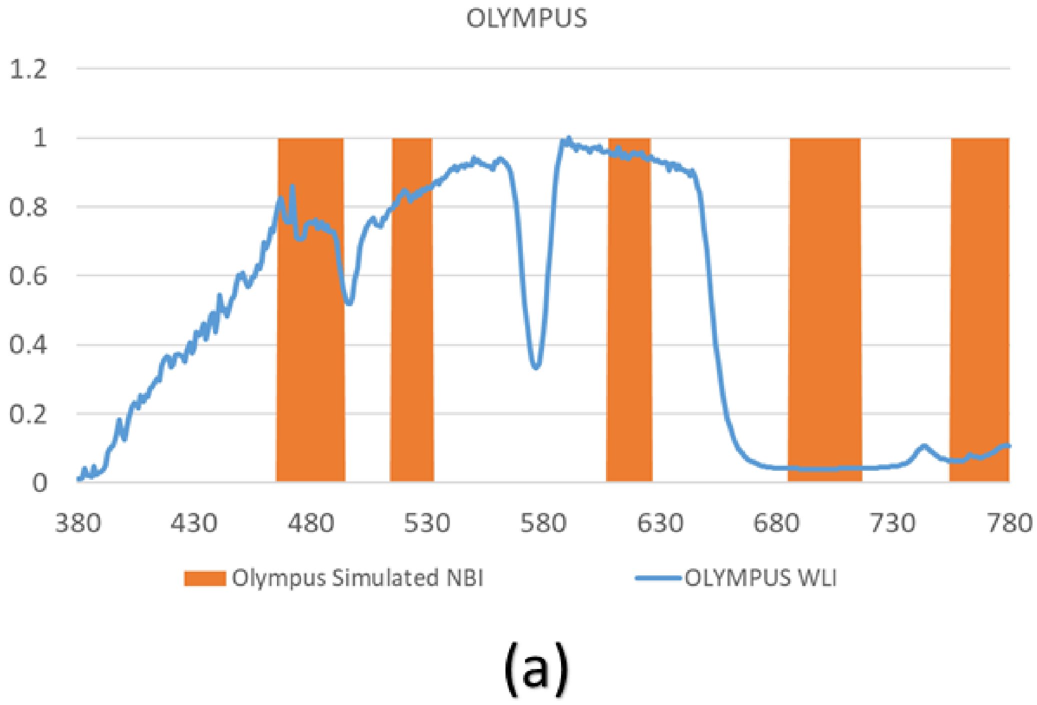

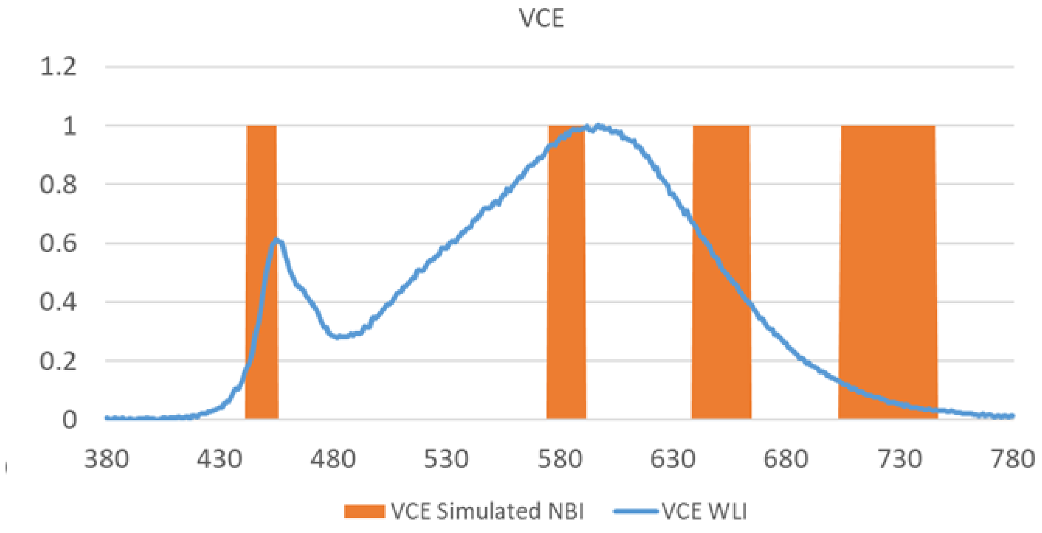
VCE
1.2
1
0.8
0.6
0.4
0.2
0
380
430
480
530
580
630
680
730
780
VCE Simulated NBI
VCE WLI

(b)

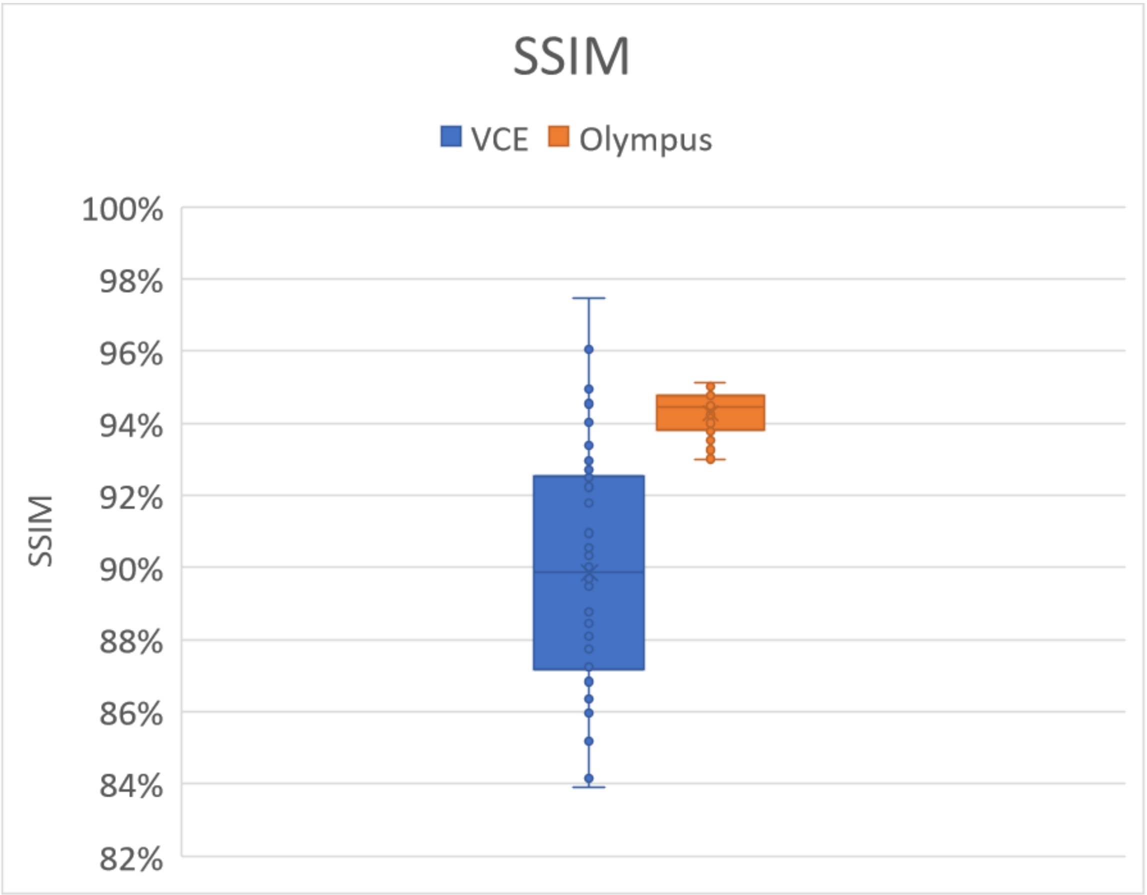
SSIM
VCE
Olympus
100%
98%
96%
94%
92%
90%
88%
86%
84%
82%
SSIM

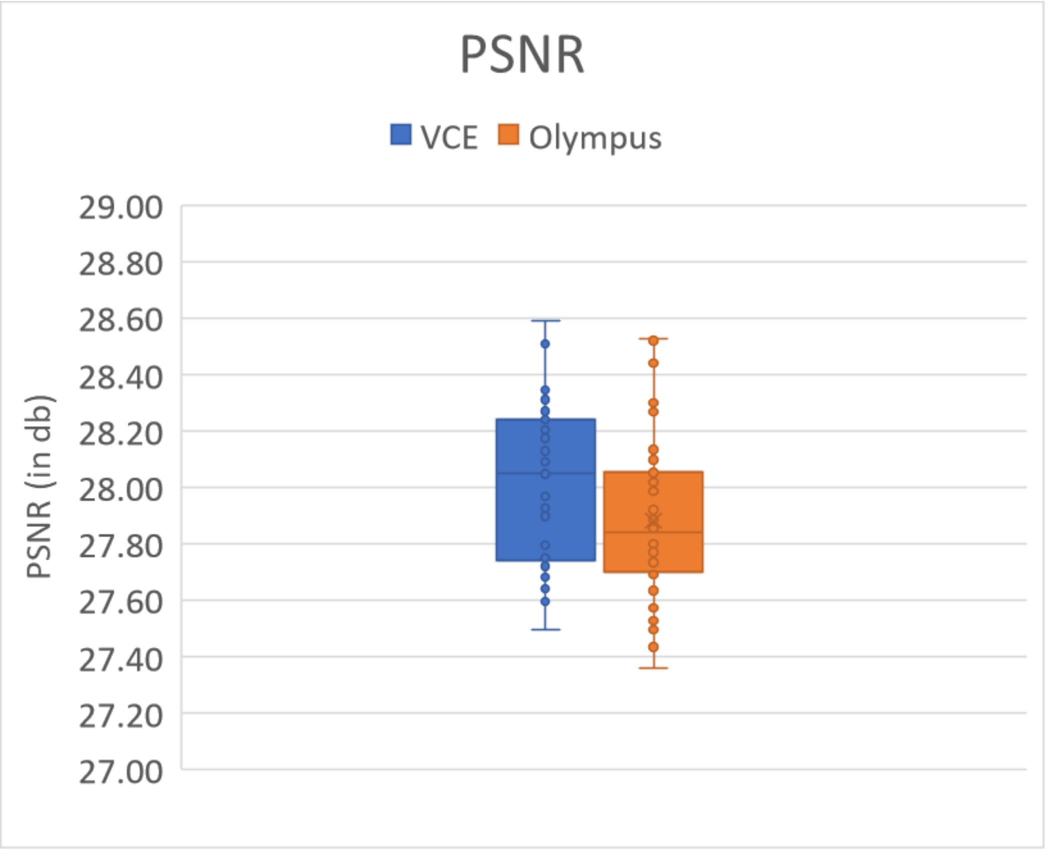

S1. Logistic Regression

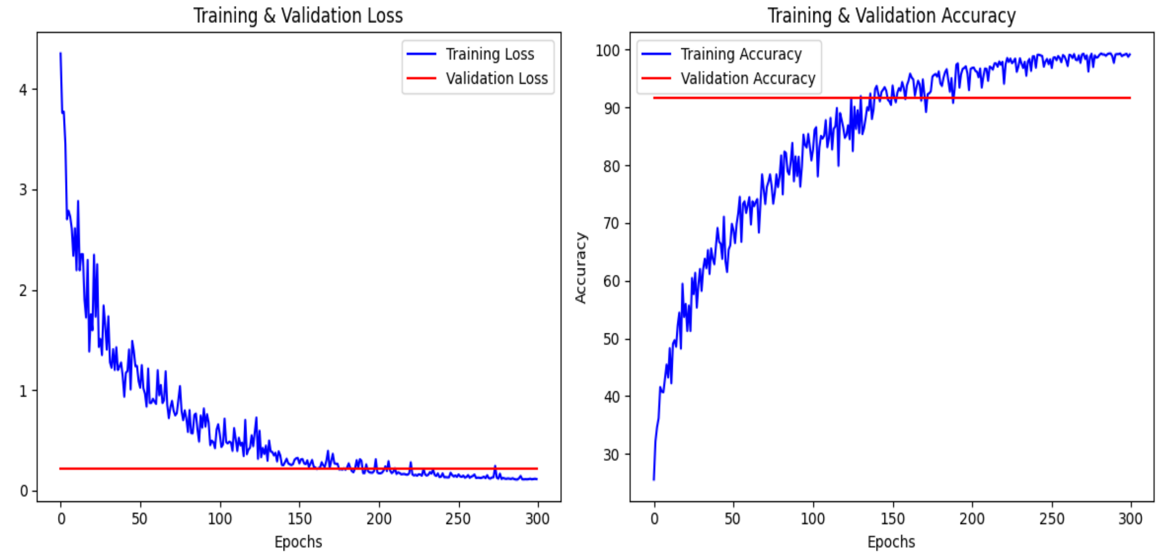

Figure S1. Training and Validation loss and accuracy for Logistic regression

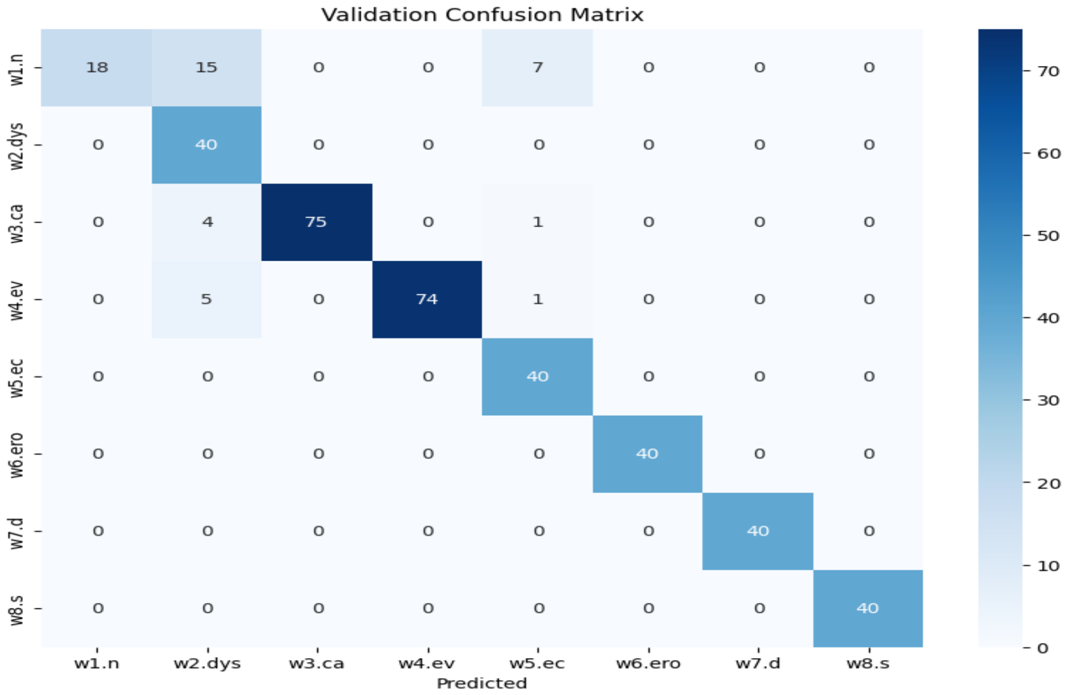

Figure S2. Confusion matrix illustrating classification performance of logistic regression on WLI dataset

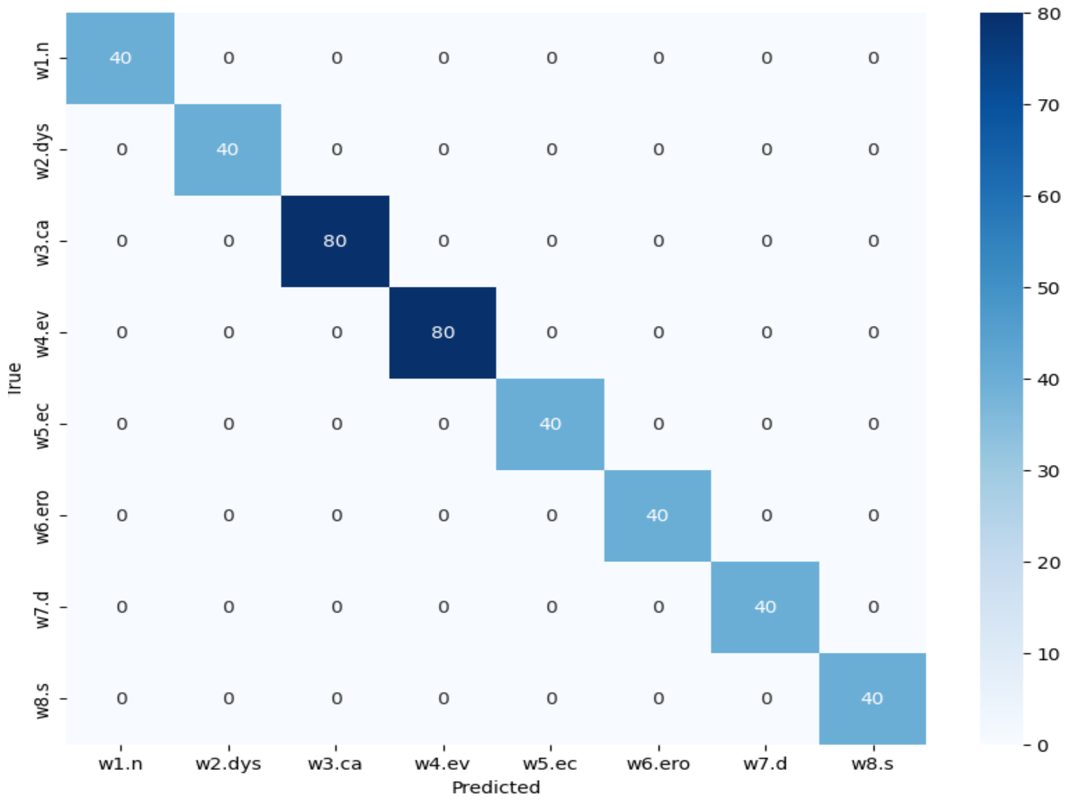

Figure S3. Confusion Matrix depicting logistic regression classification accuracy on SAVE images

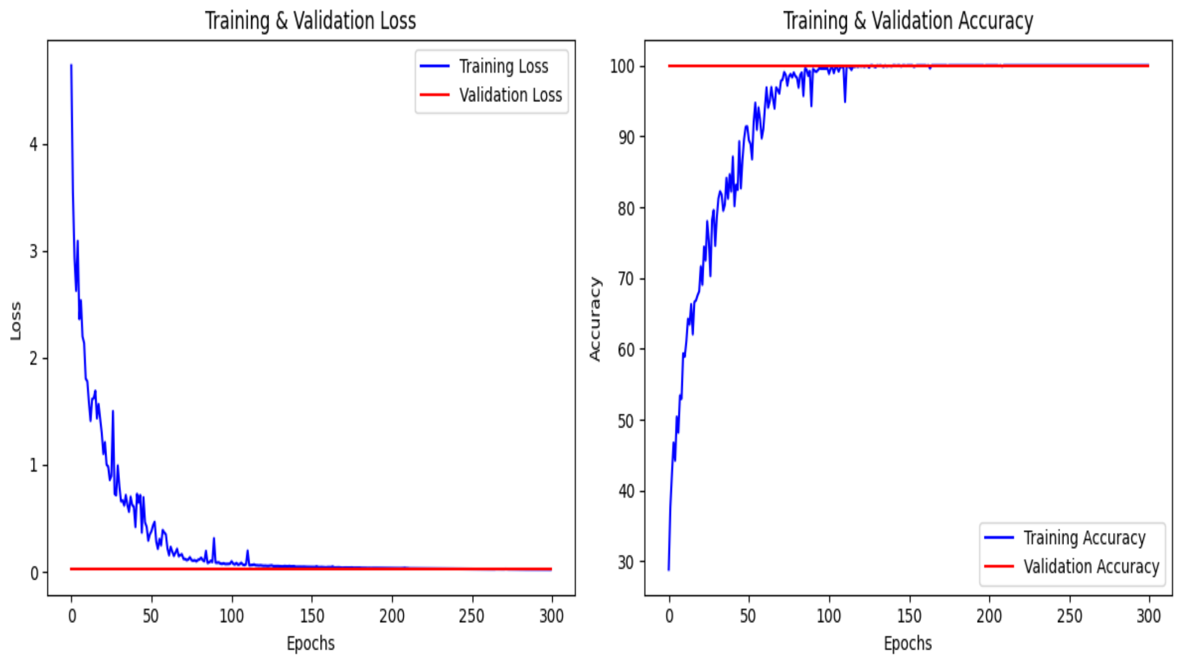

295
Figure S4. Training and Validation loss and accuracy of SAVE images for Logistic regression 296

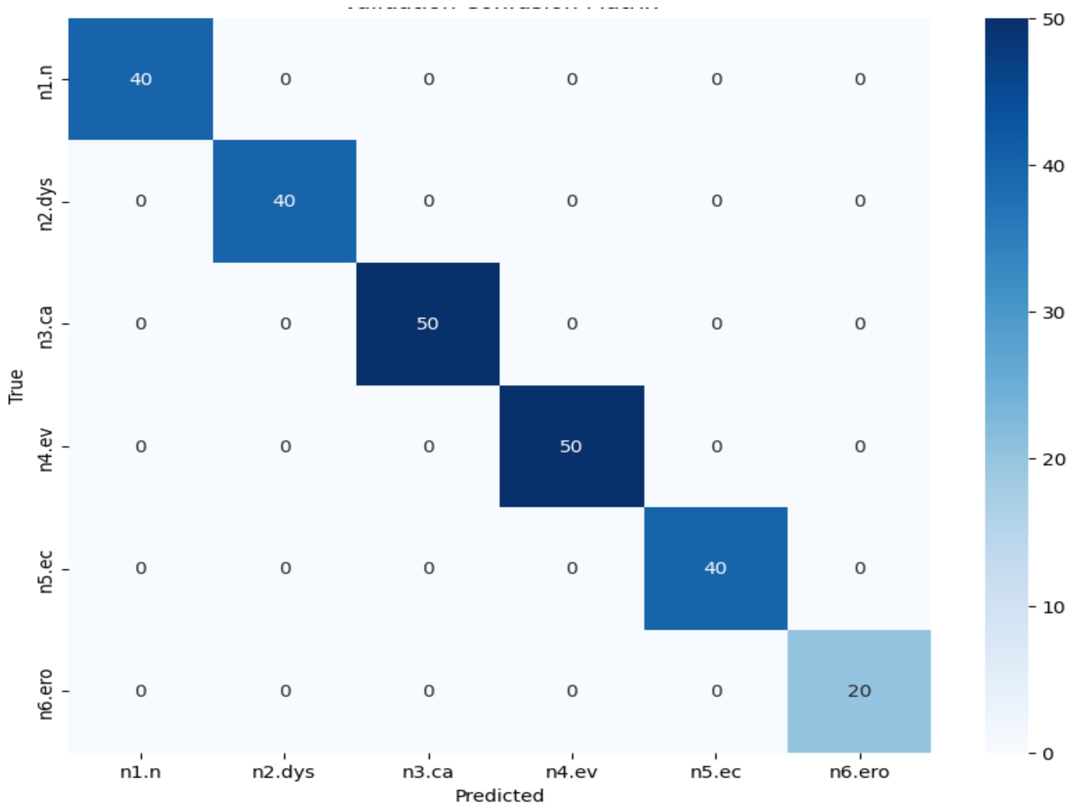

297
Figure S5. Confusion matrix for Logistic Regression performance on NBI dataset 298

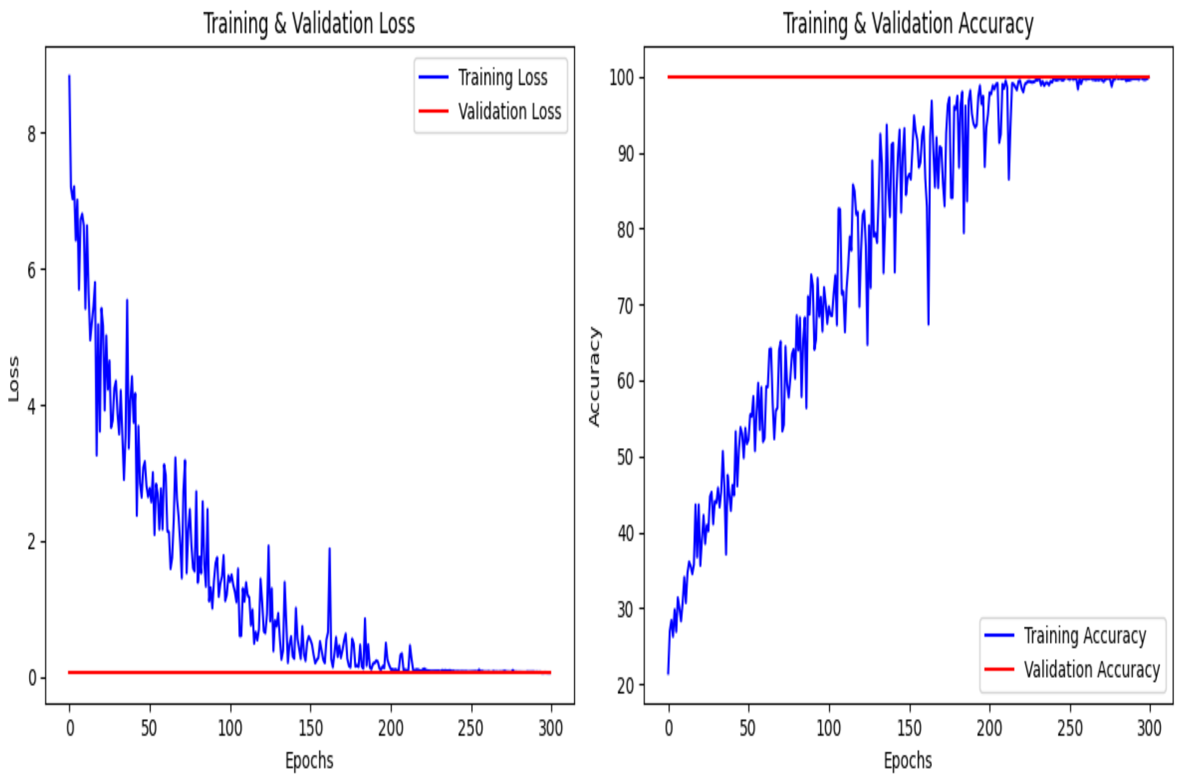

Figure S6. Training and Validation loss and accuracy of NBI images for Logistic regression

S2. VGG16

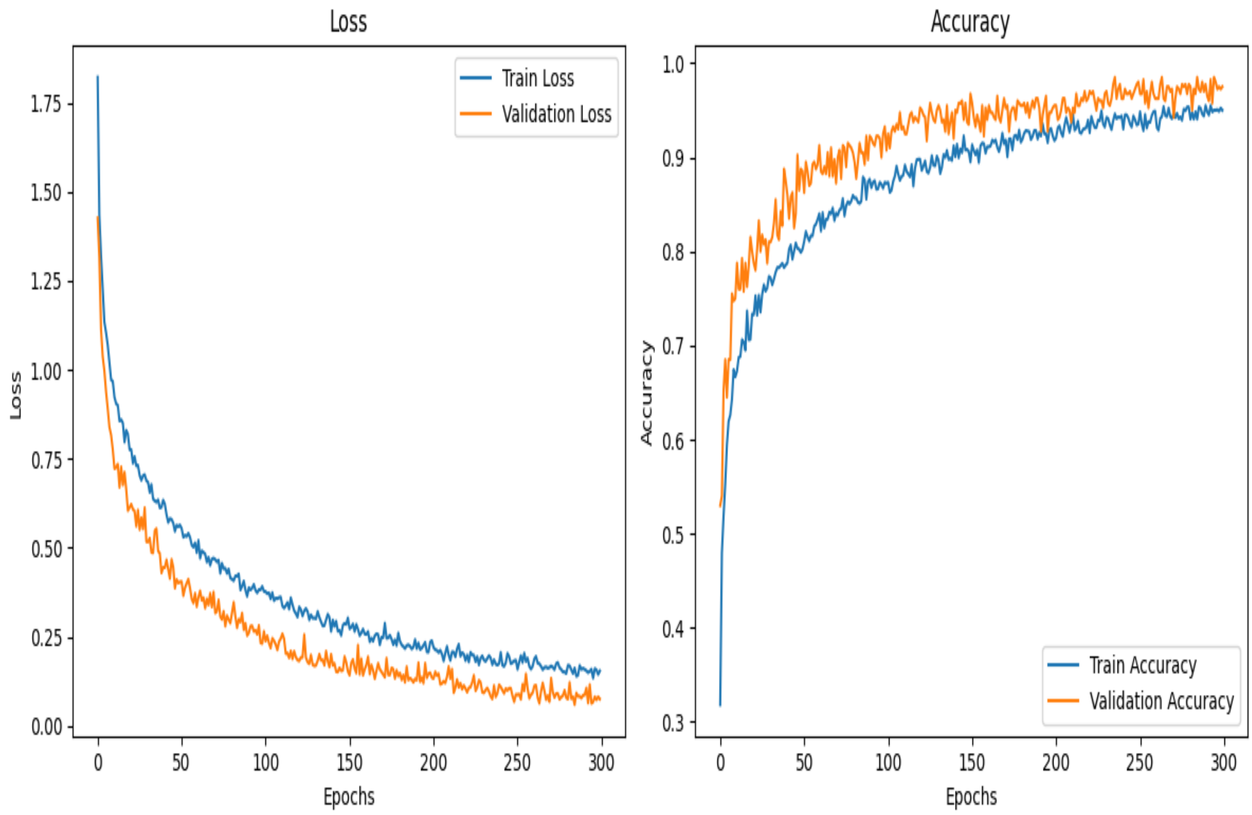

Figure S7. Training and Validation loss and accuracy of WLI images for VGG16 model

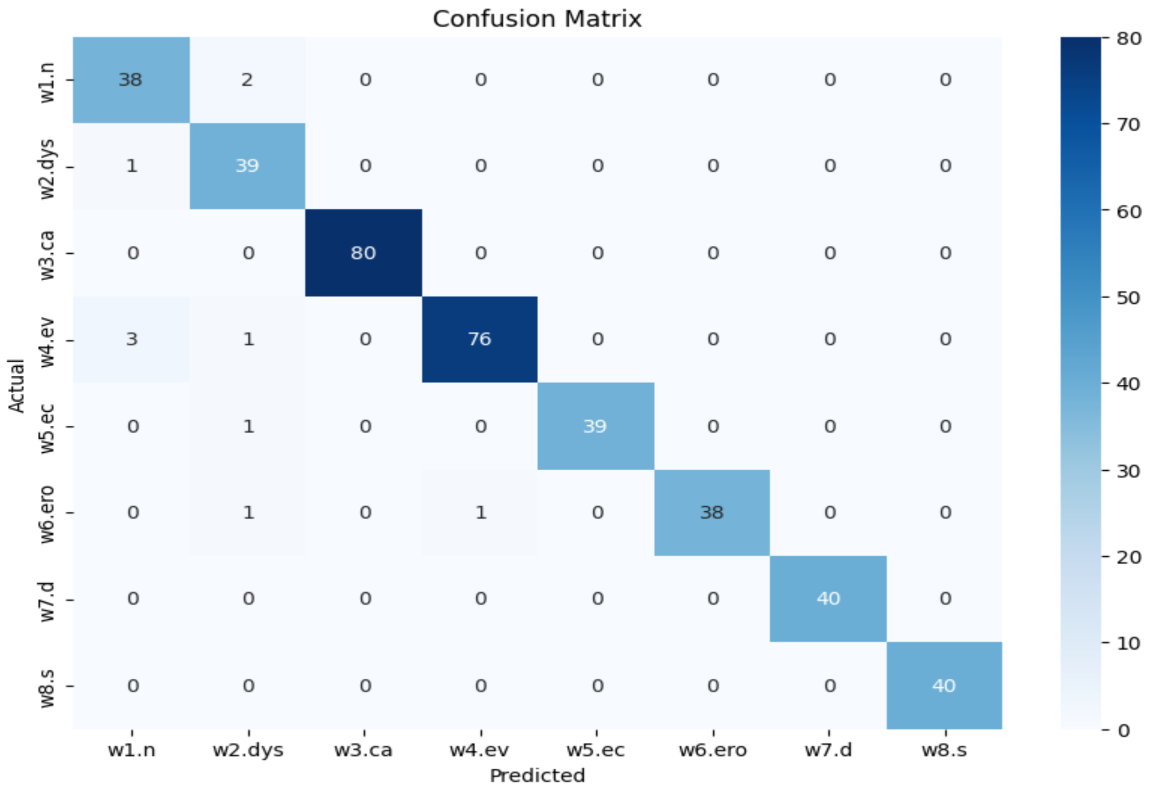

Figure S8. Confusion matrix for VGG16 classifier on WLI images indicating True Positive Rate vs. False Positive Rate

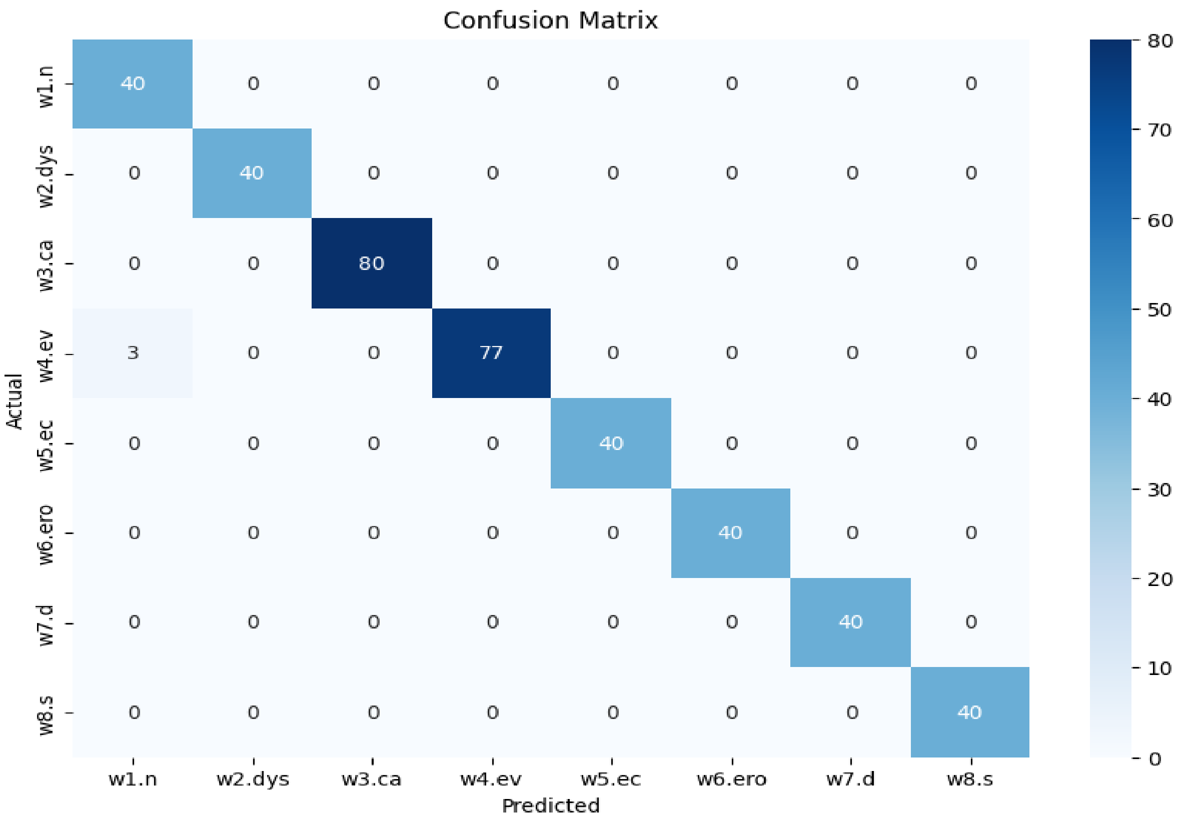

Actual \ Predicted	w1.n	w2.dys	w3.ca	w4.ev	w5.ec	w6.ero	w7.d	w8.s
w1.n	40	0	0	0	0	0	0	0
w2.dys	0	40	0	0	0	0	0	0
w3.ca	0	0	80	0	0	0	0	0
w4.ev	3	0	0	77	0	0	0	0
w5.ec	0	0	0	0	40	0	0	0
w6.ero	0	0	0	0	0	40	0	0
w7.d	0	0	0	0	0	0	40	0
w8.s	0	0	0	0	0	0	0	40

Figure S9. Confusion matrix for VGG16 model evaluated on SAVE imaging data for multiclass esophageal classification

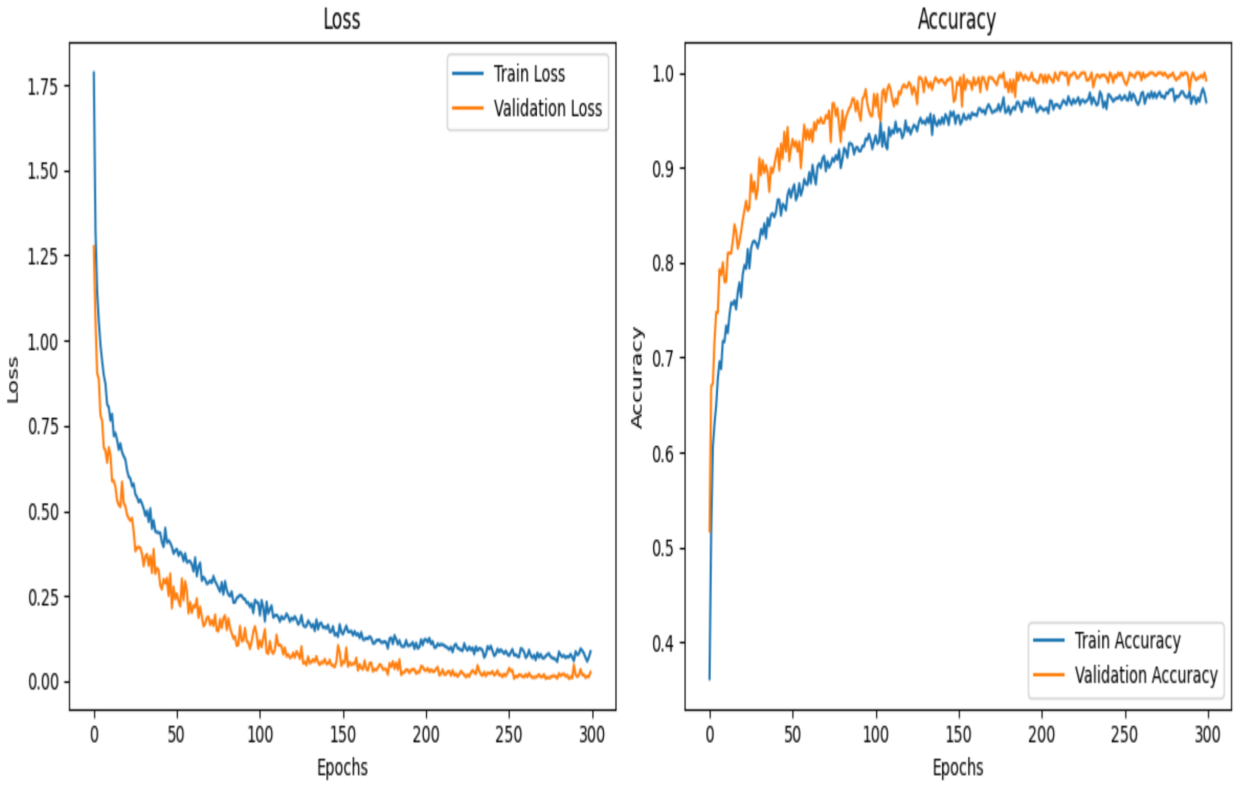

Figure S10. Training and Validation loss and accuracy of SAVE images for VGG16

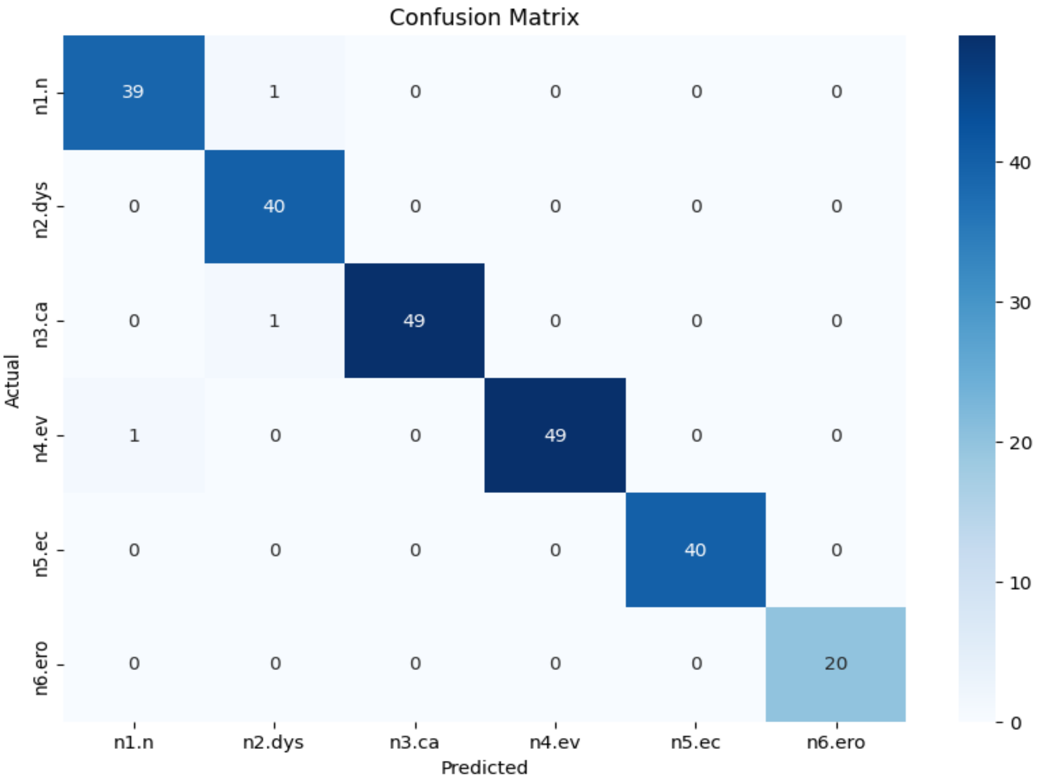

Figure S11. Confusion matrix reflecting VGG16 classification output on NBI images

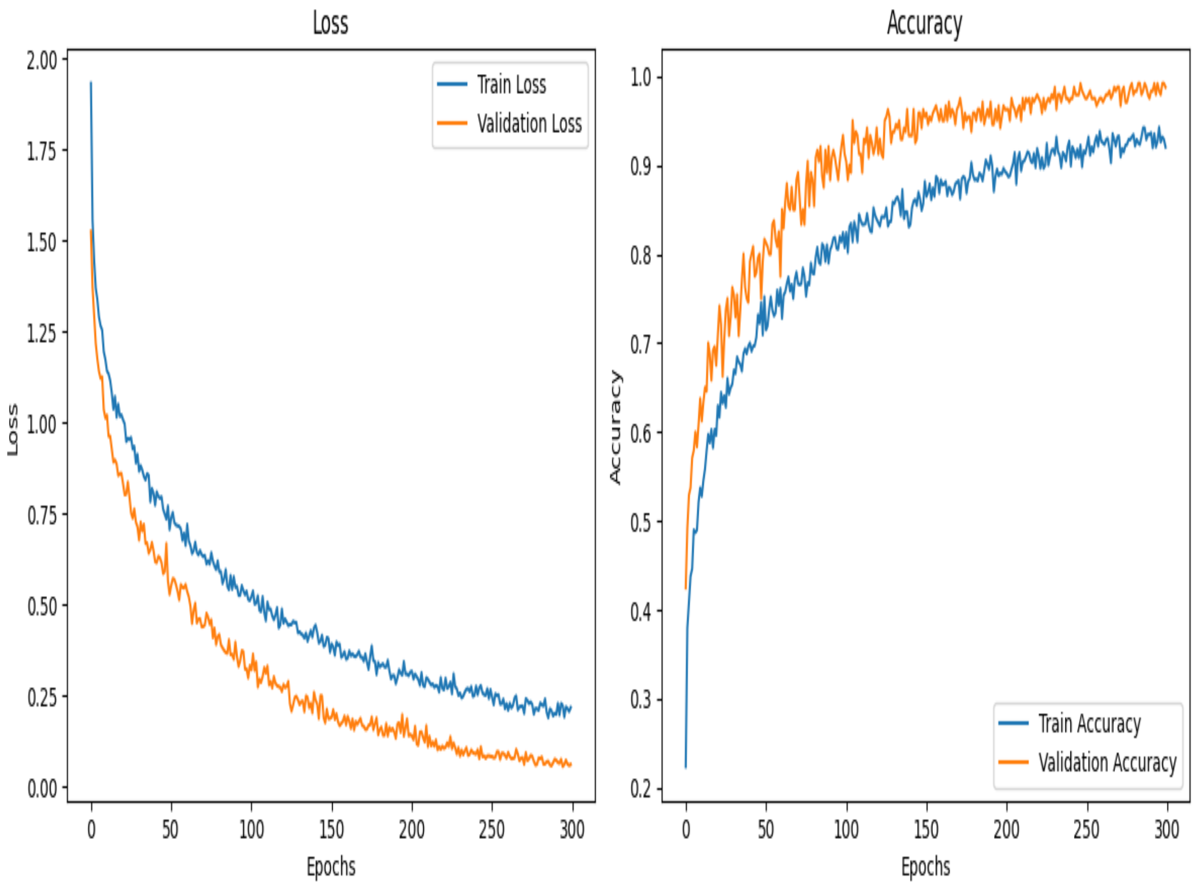

Figure S12. Training and Validation loss and accuracy of NBI images for VGG16

S3. YOLOV8 319

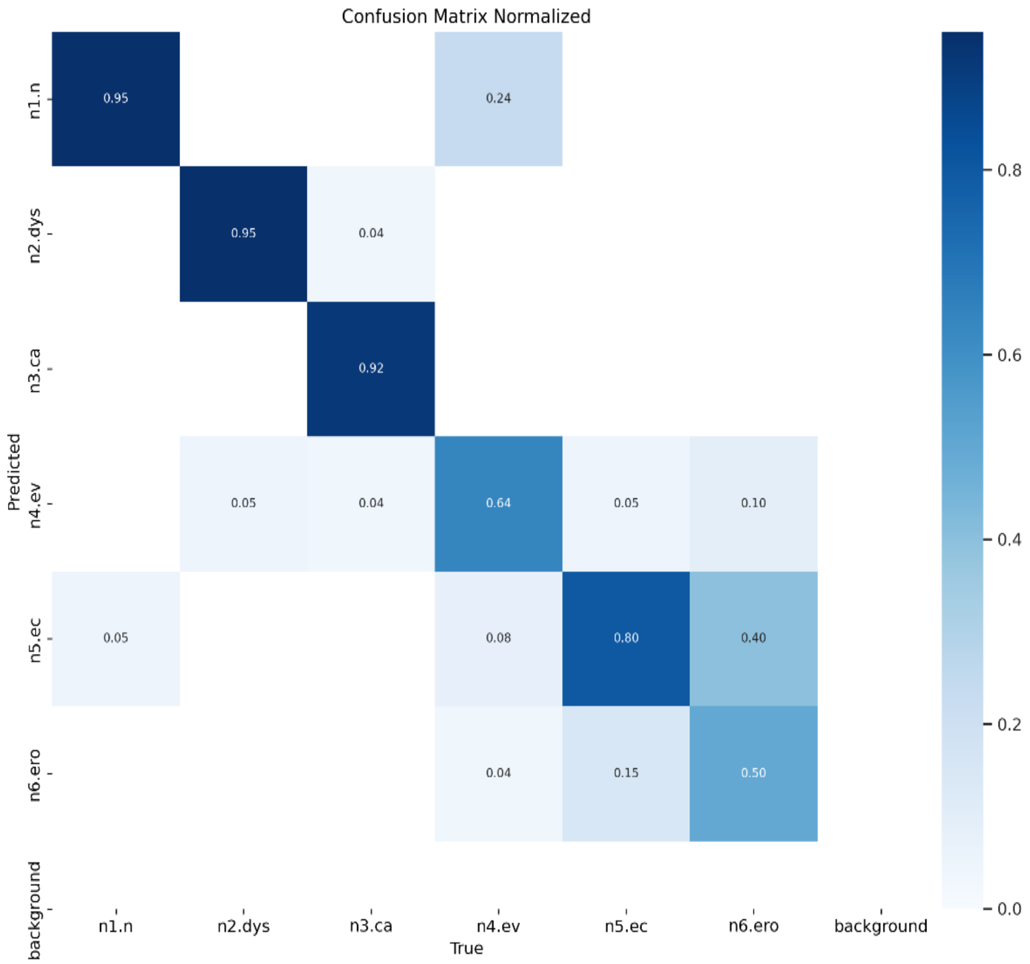

320

Figure S13. Confusion matrix for YOLOv8 object detection model on WLI images 321

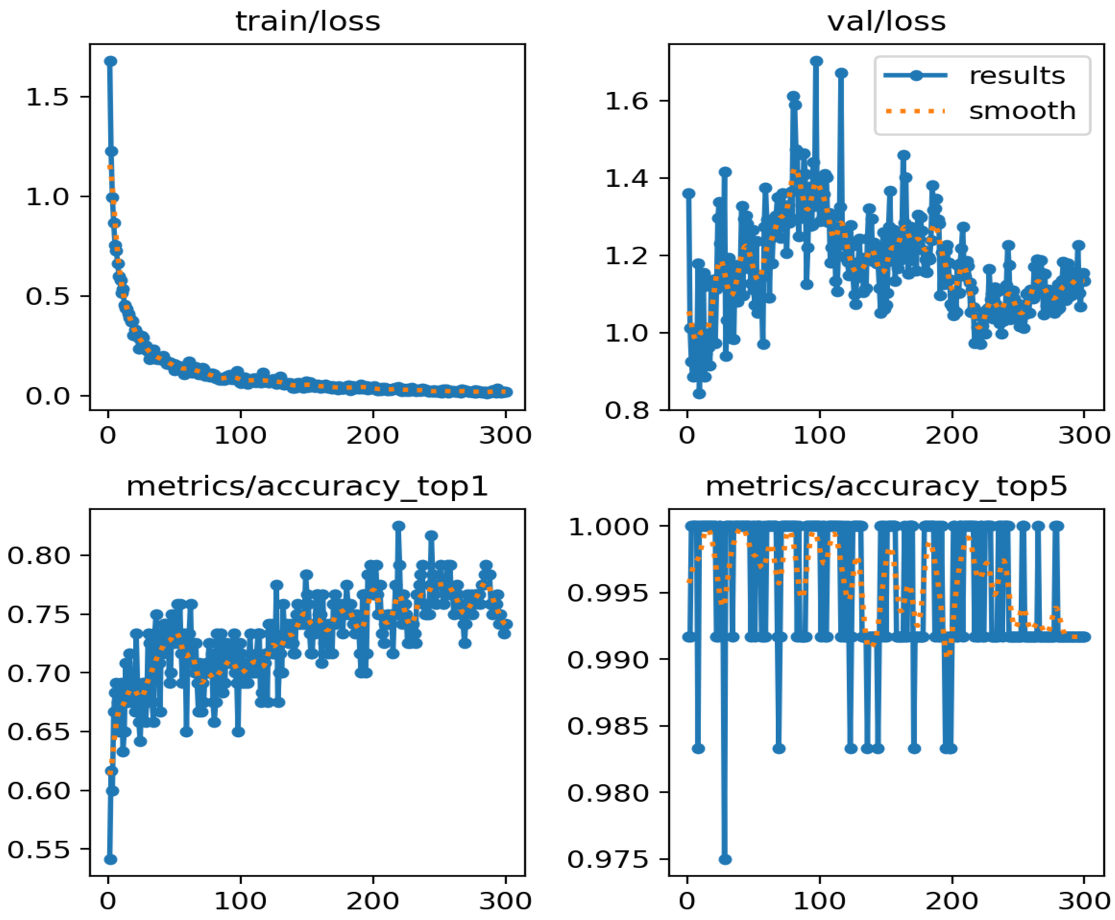

Figure S14. Detection output visualization of YOLOv8 model on SAVE images indicating Region-Based classification

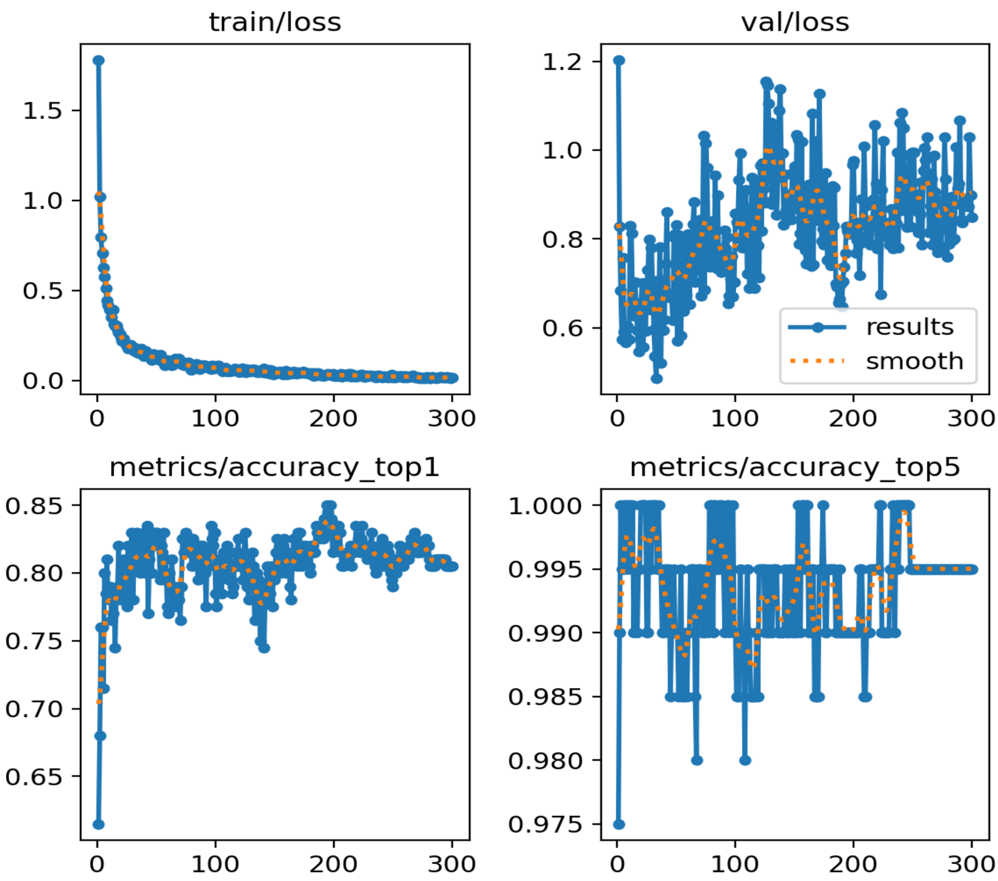

Figure S15. YOLOv8 output on NBI Images showing detection accuracy and localization of esophageal abnormalities

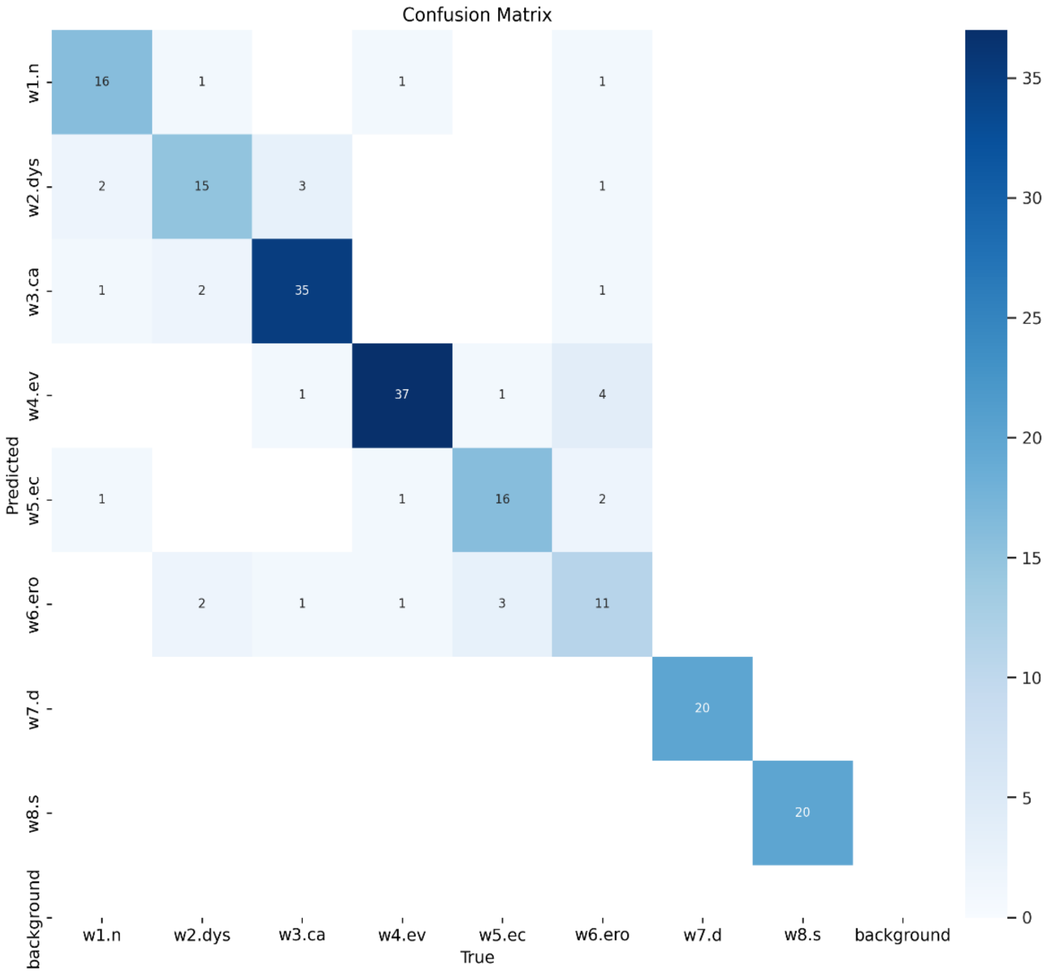

Figure S16. Confusion matrix summarizing YOLOv8 classification accuracy 329

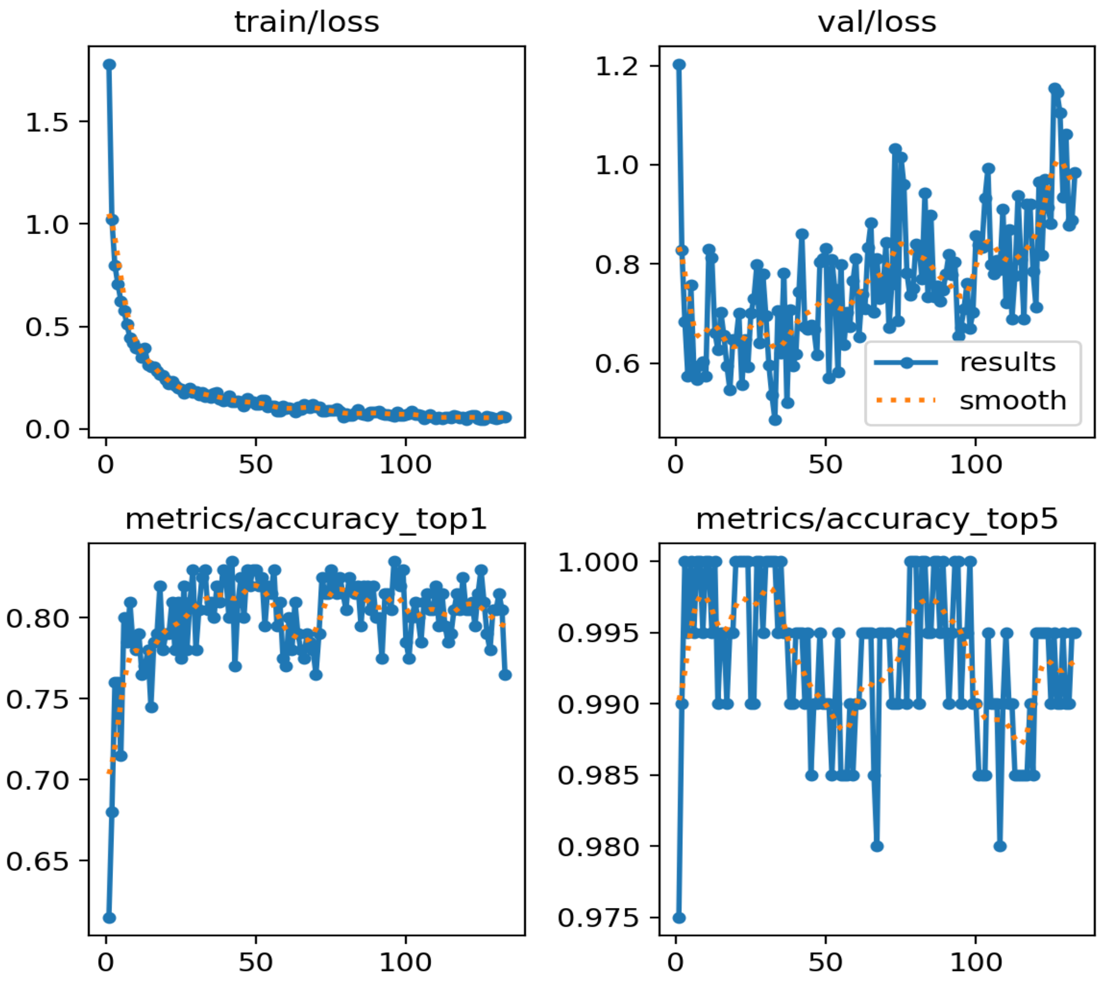

Figure S17. Precision-Recall curve of YOLOv8 model demonstrating detection trade-offs across thresholds

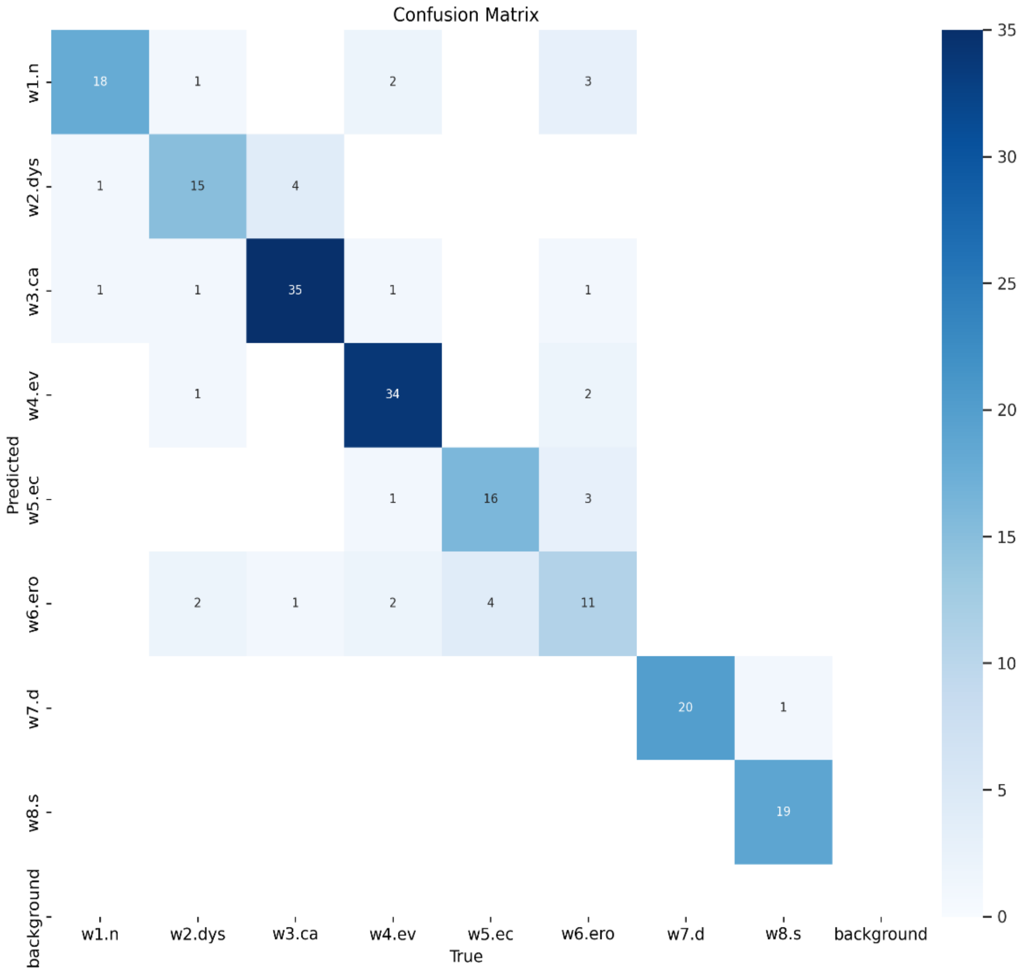

Figure S18. Confusion matrix performance of YOLOv8 model

S4. MOBILE NET V2

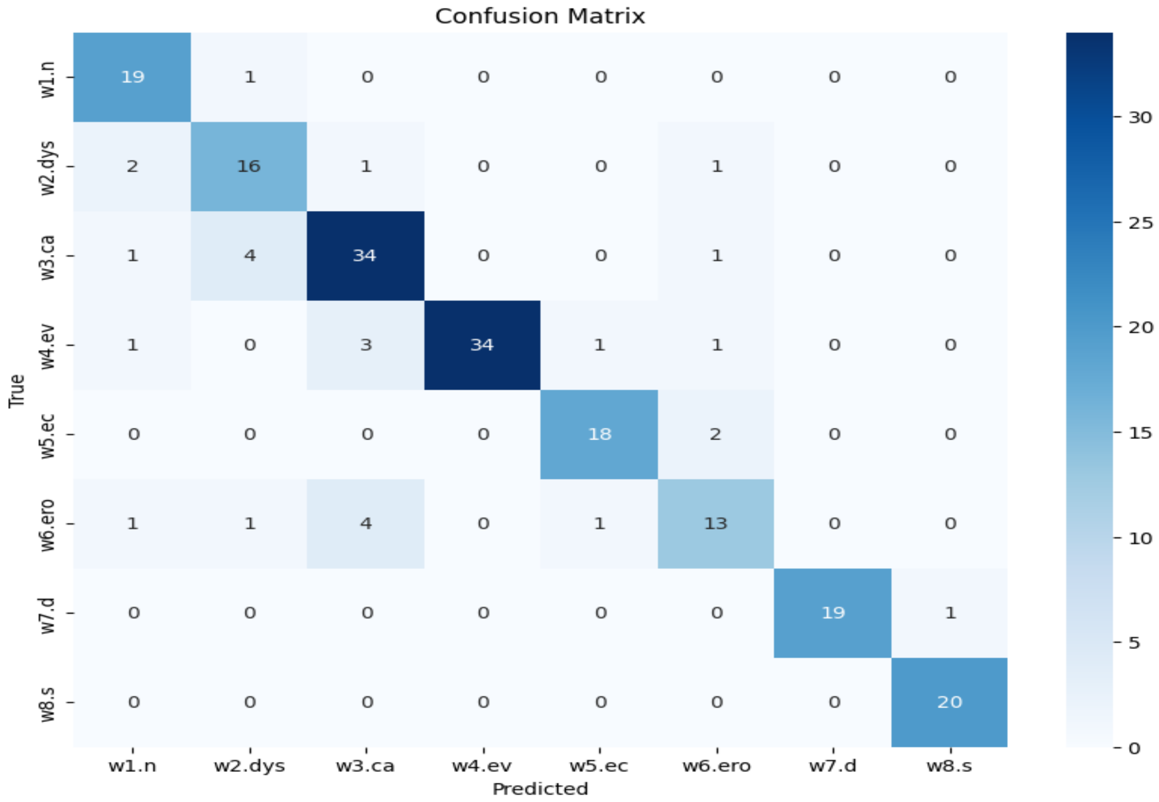

Figure S19. Confusion matrix displaying MobileNetV2 performance on WLI images for esophageal condition classification

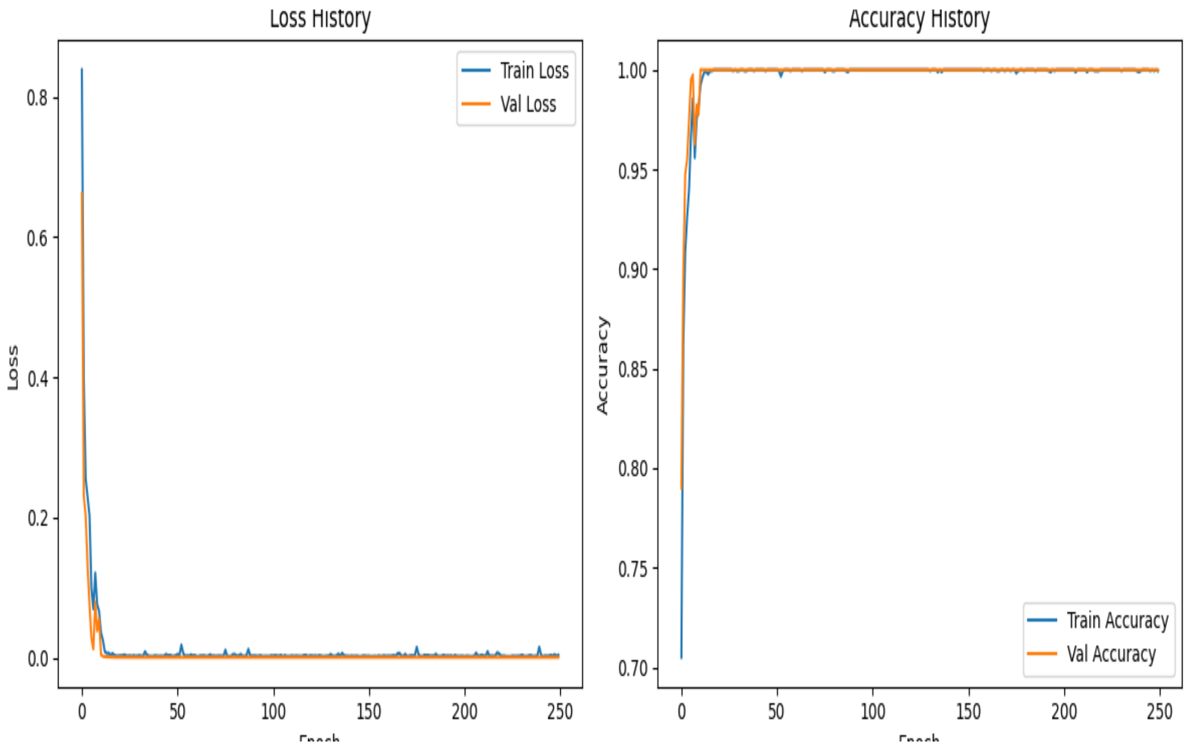

Figure S20. Training and Validation loss and accuracy of WLI images for MobileNetv2 model

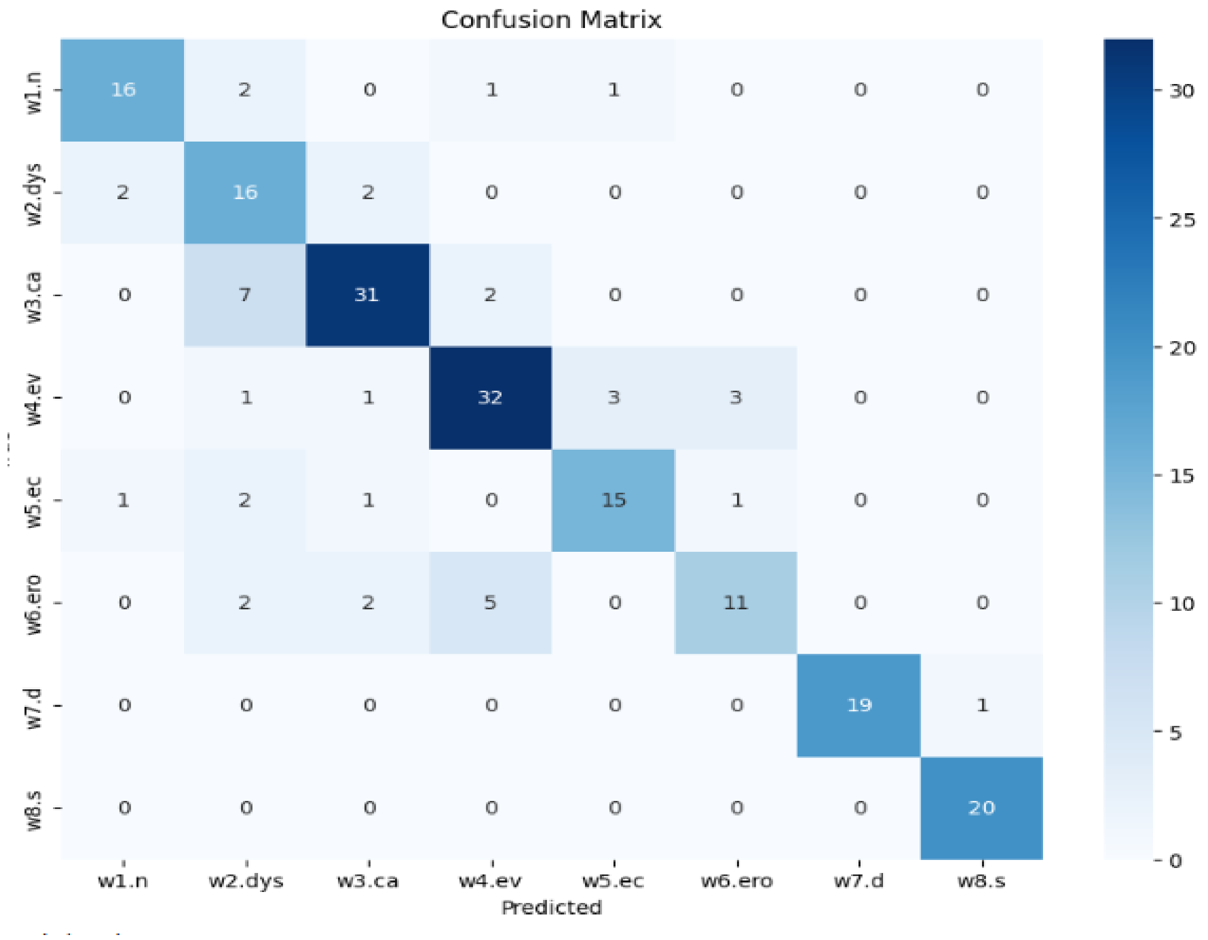

Figure S21. Confusion Matrix reflecting MobileNetV2 predictions on NBI images

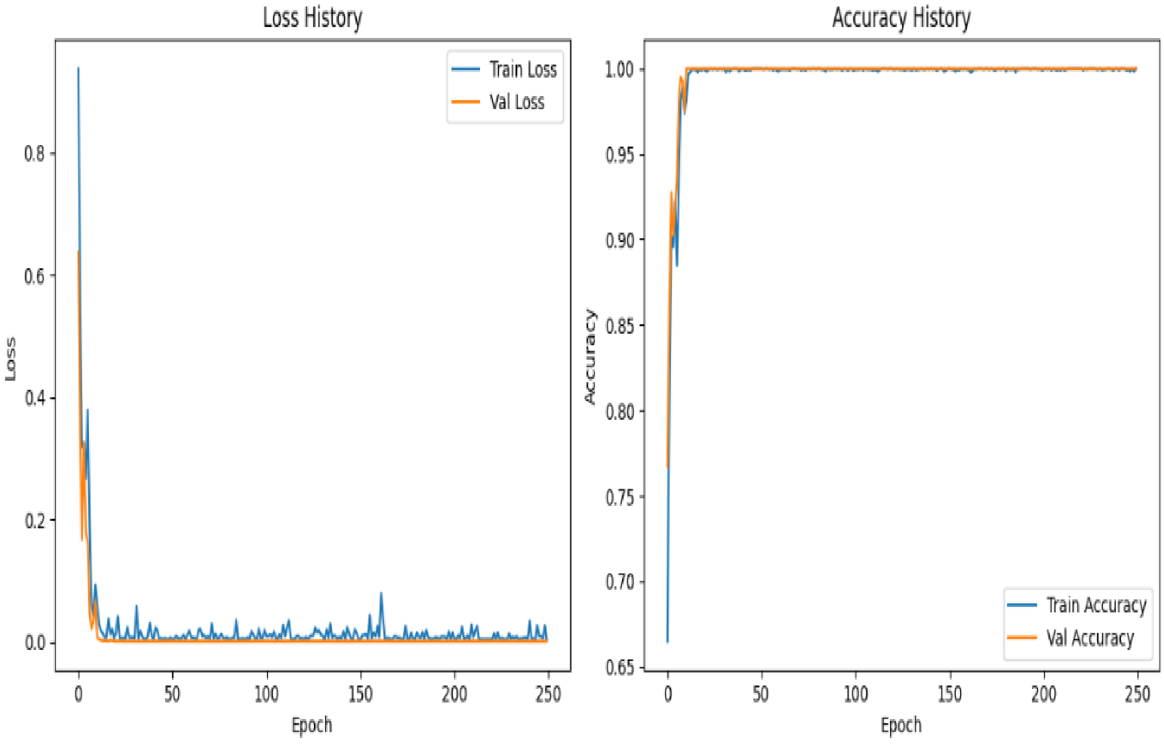

Figure S22. Training and Validation loss and accuracy of NBI images for MobileNetv2 model

S5. ALEX NET

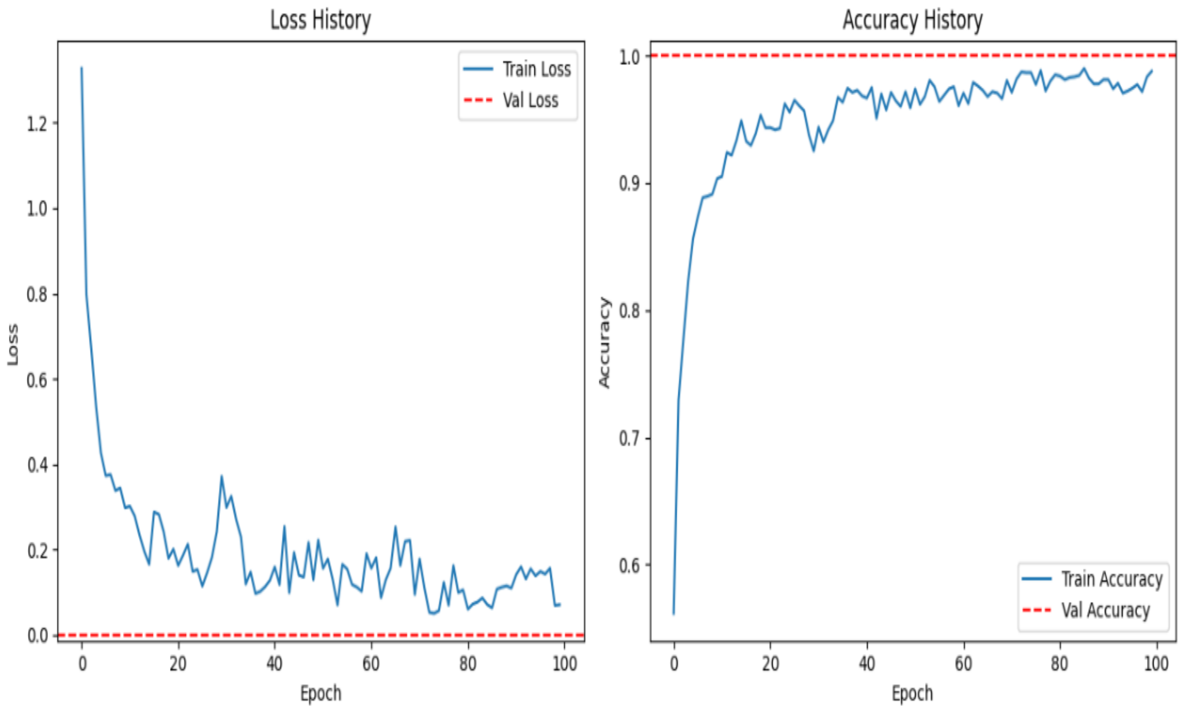

Figure S23. Training and Validation loss and accuracy of WLI images for AlexNet model

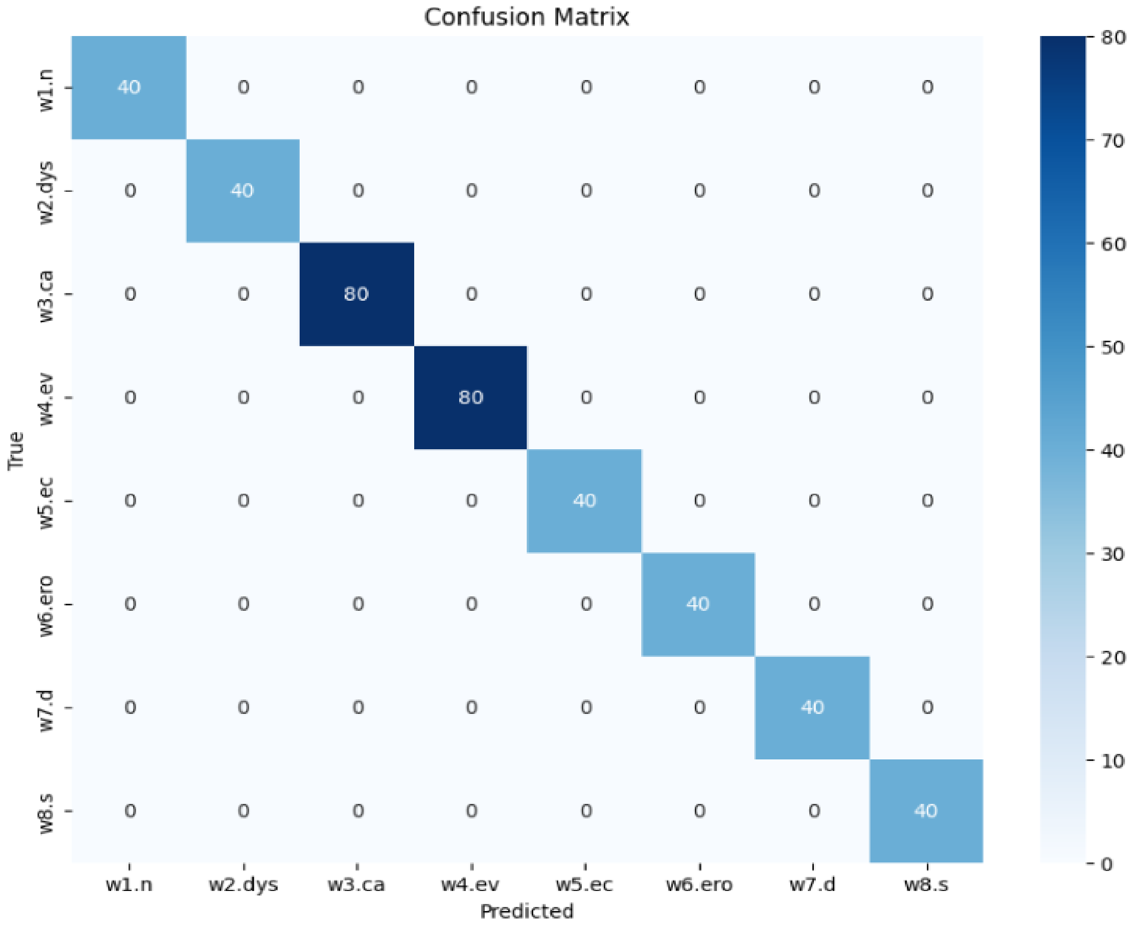

Figure S24. Confusion matrix of AlexNet classifier Applied to WLI Images with Class-Wise evaluation

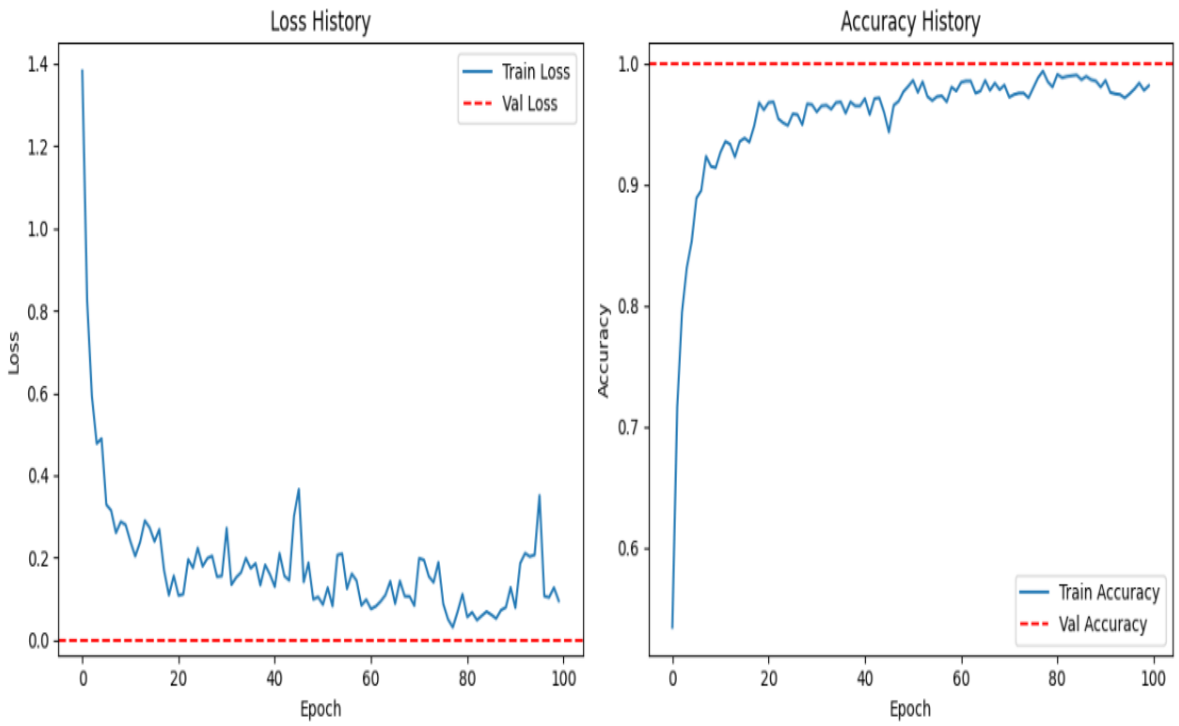

Figure S25. Training and Validation loss and accuracy of SAVE images for AlexNet model

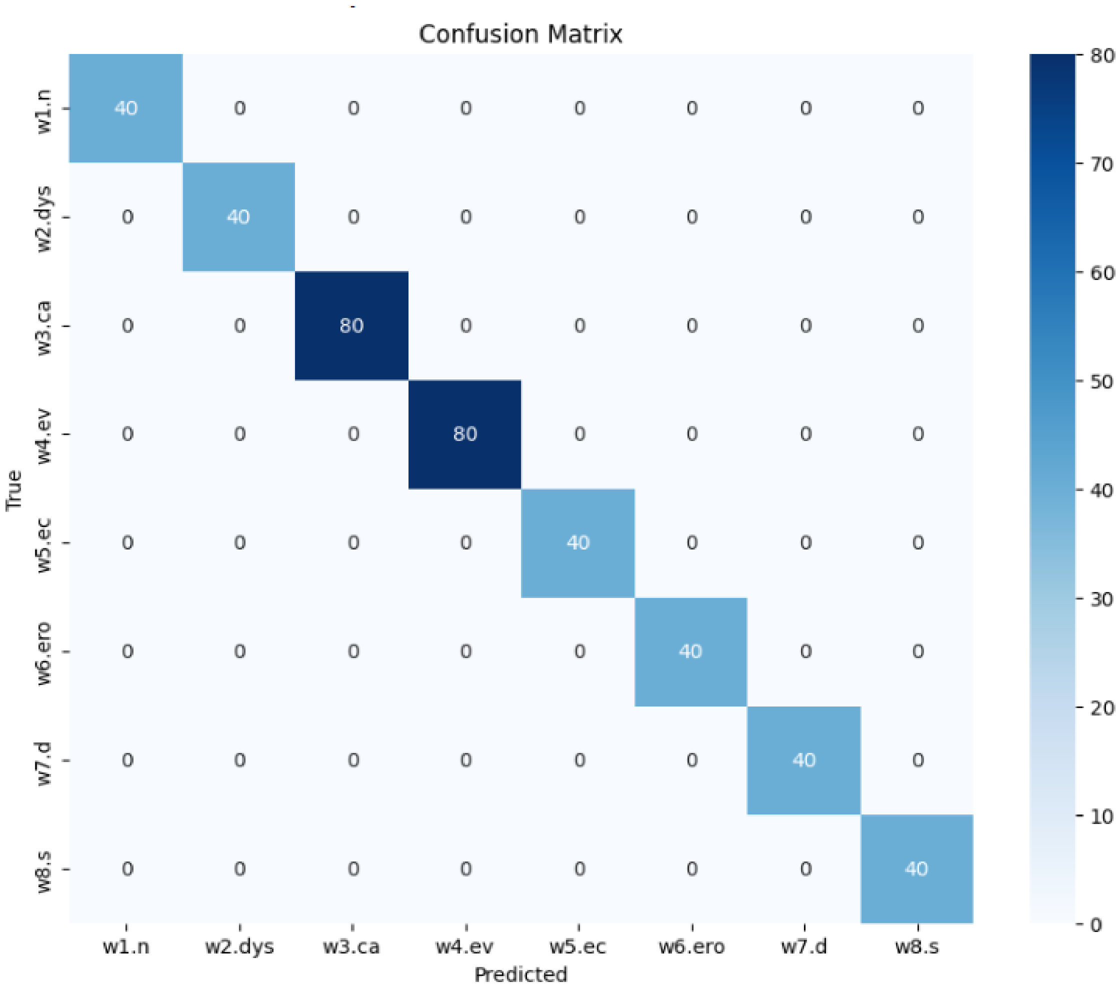

Figure S26. Confusion matrix of AlexNet classifier applied to SAVE images with Class-Wise evaluation

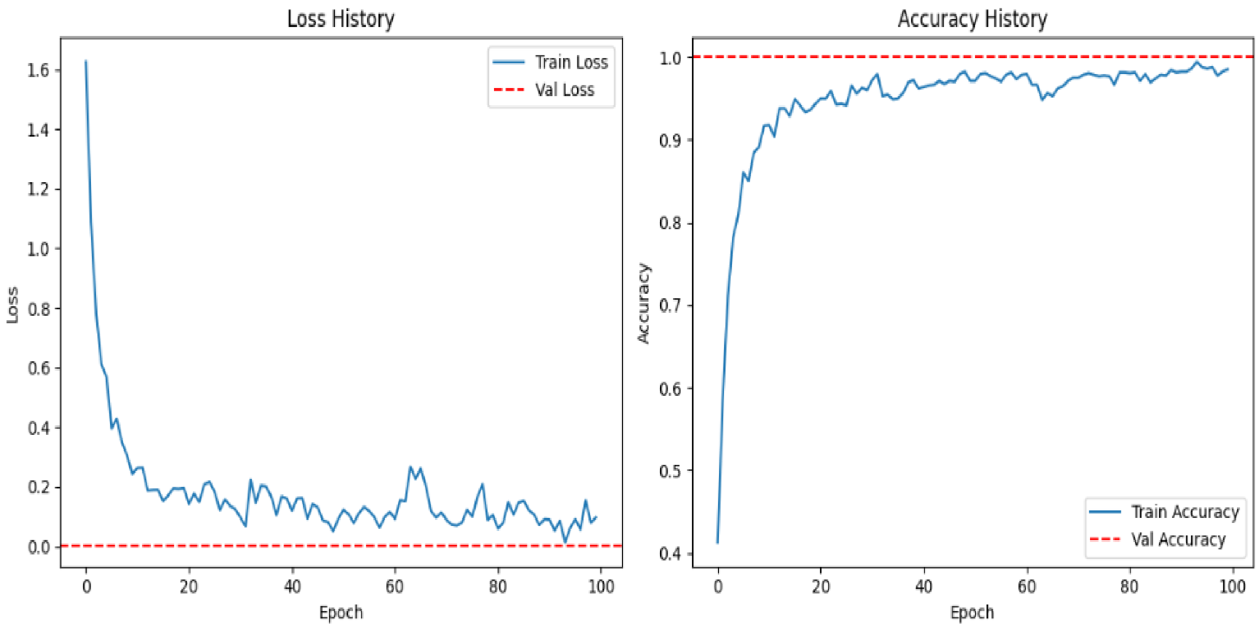

Figure S27. Training and Validation loss and accuracy of NBI images for AlexNET model

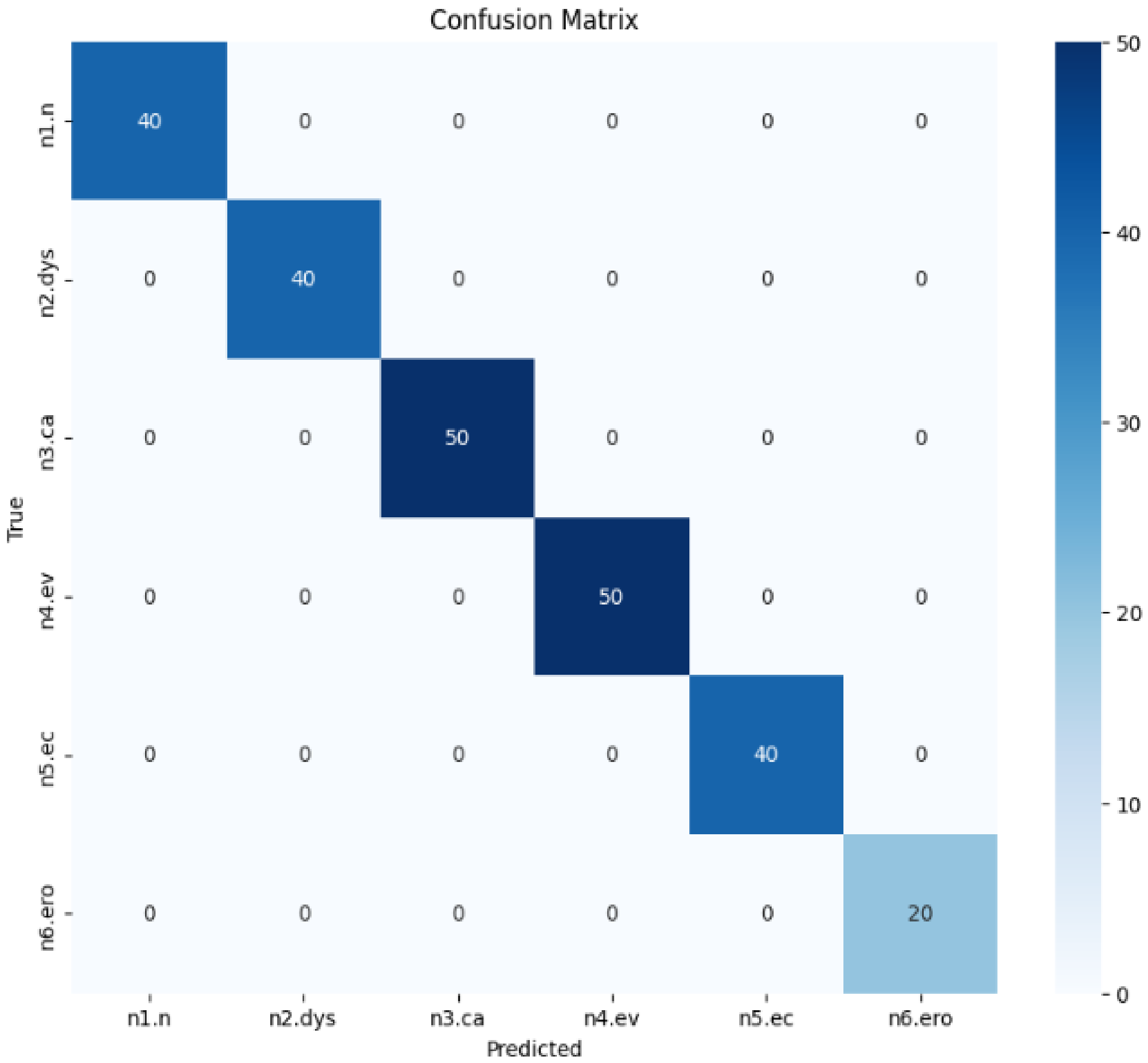

Figure S28. Confusion matrix for AlexNet applied to NBI images highlighting model prediction strengths and weaknesses

Table S3. Anova Analysis for the three imaging modalities of WLI, NBI and SAVE.

SUMMARY	Count	Sum	Average	Variance
Precision	3	293	97.6667	16.3333
Recall	3	291	97	27
F1 score	3	290	96.6667	9.3333
Accuracy	3	292	97.3333	21.3333
Precision	3	295	98.3333	1.3333
Recall	3	296	98.6667	2.3333
F1 score	3	295	98.3333	1.3333
Accuracy	3	295	98.3333	1.3333
Precision	3	249	83	16
Recall	3	244	81.3333	5.3333
F1 score	3	243	81	5.3333
Accuracy	3	248	82.6667	4.3333
Precision	3	238.75	79.5833	55.9583
Recall	3	236.87	78.9567	71.9563
F1 score	3	236.25	78.75	67.1875
Accuracy	3	241.5	80.5	33.25

Modality	Count	Sum	Average	Variance
WLI	16	1424.87	89.05438	37.12431
SAVE	16	1459	91.1875	79.7625
NBI	16	1402.5	87.65625	160.624

Source of Variation	SS	df	MS	F	P-value	F crit
Rows	3528.595	15	235.2397	11.13005	1.98E-08	2.01484
Columns	101.1984	2	50.5992	2.39403	0.10884	3.31583
Error	634.0662	30	21.1355			
Total	4263.86	47				

References

1. P. Fränti and R. Mariescu-Istodor, "Soft precision and recall," *Pattern Recognit. Lett.*, vol. 167, pp. 115–121, Mar. 2023, doi: 10.1016/j.patrec.2023.02.005.
2. H. Luan and C.-C. Tsai, "A Review of Using Machine Learning Approaches for Precision Education".
3. A. H. Villacis, S. Badruddoza, A. K. Mishra, and J. Mayorga, "The role of recall periods when predicting food insecurity: A machine learning application in Nigeria," *Glob. Food Secur.*, vol. 36, p. 100671, Mar. 2023, doi: 10.1016/j.gfs.2023.100671.
4. K. Wilkinghoff and K. Imoto, "F1-EV score: Measuring The Likelihood of Estimating a Good Decision Threshold for Semi-Supervised Anomaly Detection," in *ICASSP 2024 - 2024 IEEE International Conference on Acoustics, Speech and Signal Processing (ICASSP)*, Seoul, Korea, Republic of: IEEE, Apr. 2024, pp. 256–260. doi: 10.1109/ICASSP48485.2024.10446011.

5. M. Khalifa and M. Albadawy, "AI in diagnostic imaging: Revolutionising accuracy and efficiency," *Comput. Methods Programs Biomed. Update*, vol. 5, p. 100146, 2024, doi: 10.1016/j.cmpbup.2024.100146.
6. Z. Sun, G. Wang, P. Li, H. Wang, M. Zhang, and X. Liang, "An improved random forest based on the classification accuracy and correlation measurement of decision trees," *Expert Syst. Appl.*, vol. 237, p. 121549, Mar. 2024, doi: 10.1016/j.eswa.2023.121549.

[7] S. Dreiseitl and L. Ohno-Machado, "Logistic regression and artificial neural network classification models: a methodology review," *J. Biomed. Inform.*, vol. 35, no. 5–6, pp. 352–359, Oct. 2002, doi: 10.1016/S1532-0464(03)00034-0.

[8] R. W. Issitt, M. Cortina-Borja, W. Bryant, S. Bowyer, A. M. Taylor, and N. Sebire, "Classification Performance of Neural Networks Versus Logistic Regression Models: Evidence From Healthcare Practice," *Cureus*, Feb. 2022, doi: 10.7759/cureus.22443.

[9] Z.-P. Jiang, Y.-Y. Liu, Z.-E. Shao, and K.-W. Huang, "An Improved VGG16 Model for Pneumonia Image Classification," *Appl. Sci.*, vol. 11, no. 23, p. 11185, Nov. 2021, doi: 10.3390/app112311185.

[10] R. Vaghela *et al.*, "Land Cover Classification for Identifying the Agriculture Fields Using Versions of YOLO V8," *IEEE J. Sel. Top. Appl. Earth Obs. Remote Sens.*, vol. 18, pp. 8672–8684, 2025, doi: 10.1109/JSTARS.2025.3547058.

[11] Xingping T., Baijuan W., Hongxin Y., Zhengming Y., Rujia L., and Wendou W., "Research on fruit appearance detection and classification method based on YOLOv8".

[12] A. T. Khalaf and S. K. Abdulateef, "Ophthalmic Diseases Classification Based on YOLOv8," *J. Robot. Control JRC*, vol. 5, no. 2, pp. 408–415, Feb. 2024, doi: 10.18196/jrc.v5i2.21208.

[13] R. Indraswari, R. Rokhana, and W. Herulambang, "Melanoma image classification based on MobileNetV2 network," *Procedia Comput. Sci.*, vol. 197, pp. 198–207, 2022, doi: 10.1016/j.procs.2021.12.132.

Disclaimer/Publisher's Note: The statements, opinions and data contained in all publications are solely those of the individual author(s) and contributor(s) and not of MDPI and/or the editor(s). MDPI and/or the editor(s) disclaim responsibility for any injury to people or property resulting from any ideas, methods, instructions or products referred to in the content.
